# Health Co-Benefits of Environmental Changes in the Context of Carbon Peaking and Carbon Neutrality in China

**DOI:** 10.34133/hds.0188

**Published:** 2024-10-02

**Authors:** Feifei Zhang, Chao Yang, Fulin Wang, Pengfei Li, Luxia Zhang

**Affiliations:** ^1^ National Institute of Health Data Science at Peking University, Health Science Center of Peking University, Beijing 100191, China.; ^2^ Institute of Medical Technology, Health Science Center of Peking University, Beijing 100191, China.; ^3^ Renal Division, Department of Medicine, Peking University First Hospital, Peking University Institute of Nephrology, Beijing 100034, China.; ^4^ Research Units of Diagnosis and Treatment of Immune-Mediated Kidney Diseases, Chinese Academy of Medical Sciences, Beijing 100034, China.; ^5^ Advanced Institute of Information Technology, Peking University, Hangzhou 311215, China.

## Abstract

Importance: Climate change mitigation policies aimed at limiting greenhouse gas (GHG) emissions would bring substantial health co-benefits by directly alleviating climate change or indirectly reducing air pollution. As one of the largest developing countries and GHG emitter globally, China’s carbon-peaking and carbon neutrality goals would lead to substantial co-benefits on global environment and therefore on human health. This review summarized the key findings and gaps in studies on the impact of China’s carbon mitigation strategies on human health. Highlights: There is a wide consensus that limiting the temperature rise well below 2 °C would markedly reduce the climate-related health impacts compared with high emission scenario, although heat-related mortalities, labor productivity reduction rates, and infectious disease morbidities would continue increasing over time as temperature rises. Further, hundreds of thousands of air pollutant-related mortalities (mainly due to PM_2.5_ and O_3_) could be avoided per year compared with the reference scenario without climate policy. Carbon reduction policies can also alleviate morbidities due to acute exposure to PM_2.5_. Further research with respect to morbidities attributed to nonoptimal temperature and air pollution, and health impacts attributed to precipitation and extreme weather events under current carbon policy in China or its equivalent in other developing countries is needed to improve our understanding of the disease burden in the coming decades. Conclusions: This review provides up-to-date evidence of potential health co-benefits under Chinese carbon policies and highlights the importance of considering these co-benefits into future climate policy development in both China and other nations endeavoring carbon reductions.

## Introduction

Over the recent decades, developing nations have experienced rapid economic development by consuming large amount of fossil fuels. The accompanying emissions of greenhouse gases (GHGs; predominantly CO_2_) and air pollutants have caused devastating health issues [1]. For example, exposure to nonoptimal temperature under the context of climate change caused 3.5 deaths per million population and a population attributable fraction (PAF) of 4.7% in China in 2019 [2]. Meanwhile, as the largest global CO_2_ emitter, China has experienced severe air pollution problem and accounted for 27% (1.8 million) air pollution-related mortalities in the world in 2019 [3,4]. In order to control the rising health risk from greenhouse effect (especially in developing nations), urgent global effort is therefore needed to be initiated to control GHG emissions and to develop a sustainable green economy.

As the direct effect of GHG emissions, the global mean temperature has increased by 1.2 °C compared to preindustrial era [1]. Therefore, the goal of GHG emission control has been transformed to temperature rise control. At the Conference of Parties 21 (COP21) on 2015 December 12, nations across the globe reached the Paris Agreement, which aims to cap the increase in global average temperatures to well below 2 °C and ideally to no more than 1.5 °C compared with preindustrial levels [1,5]. To realize this goal, nations are recommended to reach a global peak of GHGs soon and to achieve carbon neutrality by around 2050 [1]. In preparation for the COP21, China made plans detailing how much it would reduce their GHG emissions and achieve the goal of carbon peaking by 2030 [3], which is known as China’s Nationally Determined Contribution (NDC). Further, on September 2020 at a UN General Assembly, China declared its intention to progressively lower CO_2_ emissions following the expected peak in 2030, with the ultimate goal of attaining carbon neutrality by 2060 [6].

There is emerging evidence that decarbonizing the world’s largest energy economy would lead to substantial co-benefits on global environment and therefore on human health—via alleviating climate change and reducing air pollution [5]. Air pollutants and GHGs share emission sources, mostly from the fossil fuel combustion; climate policies targeting GHGs would therefore reduce emissions of air pollutants simultaneously [3]. Several simulation studies have calculated the potential health benefits under the latest carbon policies in China, however with some generating substantial differences in the estimates. In this narrative review, we consolidate and assess the current evidence regarding the possible health consequences upon the attainment of carbon peaking and carbon neutrality in China and suggest the future research directions based on the current knowledge gaps. Findings from this review not only will be useful for future climate policy making in China but also provide important evidence for other nations endeavoring carbon neutrality and to better protect human well-beings.

## Pathways of Carbon Peaking and Carbon Neutrality

As a starting point, it is important to understand what carbon peaking and carbon neutrality are and the pathways to achieving them. Carbon peaking is the process that the total amount of CO_2_ emissions reaches the highest value over a specific period of time and then declines stably [6]. Carbon neutrality, also known as net-zero carbon emissions, refers to the progress that human-induced carbon emissions including both CO_2_ and non-CO_2_ GHGs are equal to the anthropogenic carbon removal over a given period of time [6].

Clearly, the attainment of carbon peaking and carbon neutrality in one of the largest developing economies faces unprecedented challenges and involves profound changes on economic and societal structure [7]. Unlike most other developed nations with a plateauing and fluctuating trend in the past few decades, China has seen a surge in its GHG emissions, especially following its accession to the World Trade Organization in 2000 [8]. This rising trend has been largely ascribed to the accelerated process of industrialization and urbanization, which relies on consuming large quantity of fossil fuels [8]. Accordingly, the multiple possible pathways to achieving carbon peaking and carbon neutrality in China are to decouple the economic growth from CO_2_ emissions (details in Supplementary Materials, p. 1–2). Main pathways include optimizing the industrial and energy mix, gradually phasing out the fossil fuels (e.g., coal), and shifting toward renewable energy (RE) (e.g., photovoltaic, wind, and hydroelectric system), improving the energy efficiency, decreasing the energy consumption, promoting low-carbon technologies and agriculture, and expanding the forest coverage (Figure and Table S1) [7,9–11]. Additionally, adopting new dietary practices and enhancing the utilization of food and agricultural waste, introducing carbon trading and tax measures, constructing resilient buildings and cities, as well as transitioning to electric-powered transportation are also effective strategies to achieve the goal of carbon neutrality [12–14].

**Figure.**
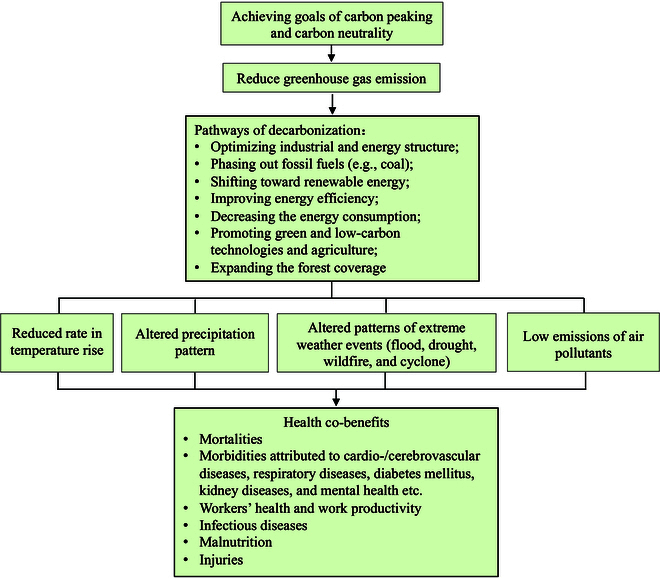
Pathways through which carbon peaking and carbon neutrality policies impact human health.

## Health Effects Related to Climate Change Driven by Carbon Policies

We followed predefined searching strategies and selection criteria for studies included in this and the following sections (Supplementary Materials, p. 1). Overall, carbon reduction policies could bring substantialhealth co-benefits via several pathways (Figure) [5,15]. Simulation studies calculated climate-related health impacts under a series of carbon emission scenarios—RCP (Representative Concentration Pathways) or SRES (Special Report on Emissions Scenarios) (Table). RCP1.9 and RCP2.6 are the representative scenarios that aim to limit global temperature rise by 2100 to below 1.5 or 2 °C above preindustrial levels, which are in line with the Paris Agreement and carbon neutrality. Several recent studies constructed climate mitigation scenarios by combining Shared Socioeconomic Pathways (SSPs) and RCPs, with climate goal scenarios of 1.5 °C, 2 °C, and NDC corresponding to SSP1-RCP1.9, SSP1-RCP2.6, and SSP2-RCP4.5, respectively [9,10,16]. Although it is unclear if carbon peaking/NDC and carbon neutrality would occur in 2030 under RCP4.5 and in 2060 under RCP1.9/2.6 in China, respectively, we linked each pair together in this review by following these studies [9,10,16], as limited studies have taken the latest Chinese carbon policies into account when projecting climate change-related health effects. Other high emission scenarios serve as controls in this review.

**Table.** Carbon emissions and temperature limit by 2100 under different carbon policies

Scenarios	Carbon concentrations or emissions	Temperature rise ^a^
Globe		
Paris Agreement/well below 2 °C goal/1.5 °C goal	Reach a net balance of zero around 2050 [1].	1.5–2 °C [1]
RCP1.9	Carbon concentrations reach 350–390 ppm (2,716–3,026 Gt) CO_2_-equiv by 2100. CO_2_ and other greenhouse gas emissions peak before 2020 and reached net zero in 2055–2075. Net-zero CO_2_ emissions are realized earlier (around 2050–2055) [108].	1–1.5 °C [109]
RCP2.6	Carbon concentrations peak at 490 ppm (3,802 Gt) CO_2_-equiv before 2100 and declines afterward [110]. CO_2_ emissions begin declining after 2020 and go to net zero by 2100 (before 2080) [108].	1.5–2 °C [109]
RCP3.4	CO_2_ emissions begin declining from 2020 and reach net zero by 2100 (after 2080), with slightly greater emissions than that of RCP2.6 [108].	2.0–2.4 °C [109]
RCP4.5	Carbon concentrations reach ~650 ppm (5,044 Gt) CO_2_-equiv (at stabilization after 2100) [110]. CO_2_ emissions peak before 2040, then decline [108].	2.5–3.0 °C [109]
RCP6.0	Carbon concentrations reach ~850 ppm (6,596 Gt) CO_2_-equiv (at stabilization after 2100) [110].	3.0–3.5 °C [109]
RCP8.5	Carbon concentrations reach >1,370 ppm (10,631 Gt) CO_2_-equiv in 2100 [110].	4.0–5.0 °C [109]
SRES B1	CO_2_ emissions reach 8.4 GtC in 2050 and 4.1 GtC by 2100 [111].	2.27 °C [104]
SRES A1B	CO_2_ emissions reach 16.8 GtC in 2050 and 14.4 GtC by 2100 [111].	3.21 °C [104]
SRES A2	CO_2_ emissions reach 15.0 GtC in 2050 and 28.5 GtC by 2100 [111].	3.83 °C [104]
China		
Carbon peaking/NDC	≈SSP2-RCP4.5: annual anthropogenic CO_2_ emissions peak by 2030 at 12.4 Gt and then decline to 9.1 Gt by 2060 [10] OR The carbon emissions peak at 10.8 Gt in 2030 and then gradually drop [87].	Above 2 °C [10]
Carbon neutrality	Carbon emissions are expected to peak in 2027, reaching ~10.6 Gt, then decrease to ~10.2 Gt by 2035, then further to ~0.6 Gt by 2060 [112] OR Between SSP1-RCP2.6 and SSP1-RCP1.9: Annual anthropogenic CO_2_ emissions reduce by 15% in 2015–2030 and further decline to 0.68 Gt by 2060, achieving net-zero CO_2_ emissions with a natural carbon sink of ∼0.7 Gt CO_2_ [10] OR The cumulative carbon budget from 2017–2060 is 150–250 Gt, the peak year is 2017–2030, the peak emission is 9.5–12Gt, and the carbon emission will increase continually before peak year and then decrease to net zero in 2060 [87].	1.5–2 °C [10,113]

SRES, Special Report on Emissions Scenarios—emissions scenarios used by the Coupled Model Intercomparison Project (CMIP3); RCP, Representative Concentration Pathways—emissions scenarios used by CMIP5; SSP, Shared Socioeconomic Pathways—scenarios added by CMIP6; NDC, nationally determined contribution

^a^ Average temperature rise compared with preindustrial level by 2100.

In this section, we mainly focused the health impacts of nonoptimal temperature under multiple RCPs. Extreme temperatures including heat waves and cold spells were incorporated as well. Precipitation was incorporated into flood/drought in the next section. The potential impact on climate-sensitive infectious diseases was summarized separately as they are correlated to multiple climatic factors.

### Nonoptimal temperature

In China, as the GHGs emit, the total population affected by heat would increase markedly even under RCP2.6 by the end of 21st century (e.g., under RCP2.6, RCP4.5, and RCP8.5 scenarios, the frequency of the warm extremes is projected to escalate by 106%, 196%, and 346%, respectively) [17]. Meanwhile, global warming is projected to not decrease or even increase the occurrence of extreme cold events in future decades [18]. Modeling studies suggest that adverse health outcomes would occur under both heat and cold temperatures [4]. Several projections for nonoptimal temperature-related deaths have been made in China (see representative studies in Table S2), particularly for heat-related mortalities as well as the net changes for both heat- and cold-related mortalities. Most projections have adopted a 3-step analysis: step 1, using a standard time series modeling strategy (e.g., distributed lag nonlinear model combined with a quasi-Poisson regression) to estimate a local exposure–response function based on historical data or previously published studies (Table S2); step 2, predicting the future temperature changes under climate change scenarios such as RCPs; step 3, predicting health effects in the future based on information from steps 1 and 2.

There is a consensus that limiting temperature rise < 2 °C substantially lessens climate-related adverse health impacts compared with high emission scenario, although compared with the historical periods future heat-related deaths would increase even under RCP2.6 (Table S2) [19], particularly during the summer [20]. Considering the urban inhabitants alone, it is projected that the incremental warming from 1.5 to 2 °C would result in an excess of 27,900 heat-related deaths in China every year [21]. The vulnerable individuals affected by future heat have been explored by cause of deaths, demographics, and regions. The heat effect will be particularly severe in people with cardiovascular and respiratory diseases [19,20], the older people [19,21,22], females [19,21], people with low educational attainment [19,21], nonurban residents [20], and people from eastern and central China [19,23–25]. For example, Chen et al. [20] found that under RCP4.5, nonaccidental mortality in rural counties of Jiangsu Province was nearly 3 times higher than that in urban counties in the summer of 2041–2045 compared with the 1981–2005 baseline. The heat effects are anticipated to be considerably exacerbated by population aging [19,24,26,27], rapid population growth [26,27], and high GHG emissions [19–21,23,27]. By contrast, adaptation to heat is expected to mitigate the excess heat mortality [21,23,26], although the extent of adaptation remains unclear [23]. Moreover, heat effects have been explored by considering the night heat or day and night heat simultaneously [the so-called compound heat extremes (CHEs)], which also favors the findings that less GHG emissions mean less heat-related deaths in the future [28,29].

Few studies projected the future cold-related mortalities in China. One study in Guangzhou found that the cold-related deaths would increase by 0 to 0.3 times in 2020s to 2080s under RCP4.5 and RCP8.5 compared with the baseline in 1980s [30]. Another nationwide study found that future cold-related deaths were expected to increase in most decades by 2100 under RCP2.6 (81.5% in 2050s and 37% in 2090s) and RCP4.5 (55.5% in 2050s and −19% in 2090s) compared to the baseline period in 1995–2005. Population aging would amplify these effects (increase by 101.1% in the 2050s and 146.2% in the 2090s) [31].

Taken the heat and cold effects together, net excess deaths attributed to climate change are projected to decrease in most studies, particularly under low carbon emission scenarios such as RCP2.6 (Table S2). This suggests that the rise in heat-related deaths attributed to climate change will be offset by the decrease in cold-related deaths [32–35]. However, there are exceptions that cold-related death reduction cannot offset the rise in heat-related health effects, which include the high population variation scenario [32,36–40], certain areas in southeast of Hu line (e.g., Beijing–Tianjin Metropolitan Region, Yangtze River Delta, Guangzhou, and Wuhan) [32], those aged ≥65 years [41], scenarios under high GHG emissions [35,41–45], and some specific causes [e.g., ischemic stroke [36], cardiovascular diseases (CVDs) [37,43], ischemic heart disease (IHD) [35,39], and external causes [35]]. Effect of adaptation can further complicate the trend; adaptability to heat would increase, whereas to cold would reduce, due to reduced exposure to cold environments [46]. One study found that when the warm adaptation was adopted, the net mortality risks became smaller [40]. However, other studies suggested that the adaptation to climate change is unlikely to reduce the net temperature-related mortalities, particularly under high GHG emissions and demographic changes [34,38,43] and for causes such as CVD and IHD [37,39].

Empirical evidence has also linked nonoptimal temperature to a series of morbidities including cardiovascular/cerebrovascular diseases, respiratory diseases, diabetes mellitus, kidney diseases, mental health and neurological diseases, as well as offspring and maternal health (Supplementary Materials, p. 2). Similar with mortalities, these morbidities would also be likely to rise in the context of climate change. Nevertheless, there have been limited studies projecting the nonoptimal temperature-related morbidities in China. One recent study estimated future morbidity burden due to compound extremes of heat and humidity (i.e., humid-heat weather) in Shanghai [47]. Assuming that there is no population change and adaptation, humid-heat-related attributable fractions (AFs) would rise by 17.5% by 2100 under SSP5-8.5 compared to the baseline in 2010–2019, which was 4 times higher than that under SSP1-RCP2.6 (4.4%), and was much higher than the AF related to heat only (9.9%). Diseases including respiratory and cardiovascular diseases were particularly sensible to the humid-heat weather [47]. By contrast, another study using data from inpatient visit claims spanning 47 cities across 28 provinces in China projected that hospital admissions under RCP8.5 in 2070–2099 showed no statistical differences compared to the reference period in 2008–2010 [48]. Available evidence from other countries suggested that morbidities of heat-related cardiovascular, cerebrovascular, and respiratory diseases, nephrolithiasis, sleep loss, and dementia would increase even under RCP2.6 (details in Supplementary Materials, p. 2–4). The net change for heat- and cold-related morbidity of myocardial infarction and low birth weight would be negligible or even negative under RCP2.6 and RCP4.5 [49,50].

Compared with the general population, workers are particularly vulnerable to nonoptimal temperature-related mortalities and morbidities (e.g., heat-related symptoms and injuries [51,52]). Moreover, exposure to high-temperature conditions subjects the human body to heat stress (so-called occupational heat strain) and further affect workers’ productivity outcomes, which would indirectly affect health effects by reducing the family income [51,52]. Several studies have projected the temperature-related work productivity in China, with one study suggesting a loss by more than 10% and 40% by 2100 under RCP2.6 and RCP8.5 compared to the baseline from 1981 to 2010, respectively [53]. Most prominent loss would be seen in workers engaging in heavy work, regions including south and east China, or lack of adaptation strategies, as well as outdoor or the so-called labor-intensive sectors encompassing construction, mining, and agriculture [53–57]. Labor productivity in indoor sectors will show a slight decrease (RCP8.5-SSP3) or even increase (RCP2.6-SSP1, RCP4.5-SSP2) due to high air-conditioning device rates in 2030–2100 [54]. Occupational injuries have been projected in countries outside of China. Fatima et al. [58] estimated that net AFs would increase under RCP4.5 and RCP8.5 in Greater Adelaide in the 2050s (versus 2010s). In China, few studies projected occupational injuries, but one recent study using the injury death data observed an upward trend in the injury mortality burden associated with CHEs between 2010 and 2090 under both RCP4.5 and RCP8.5 scenarios, with pronounced effects in southern, eastern, central, and northwestern regions of China. Under the RCP2.6 scenario, the AFs for these injuries are projected to escalate in 2030s (1.68 times) and 2060s (1.97 times), followed by a decline in 2090s (1.74 times) relative to the 2010s [59].

### Climate-sensitive infectious diseases

Infectious diseases are highly sensitive to a series of climatic factors such as temperature, humidity, and rainfall. Of them, vector-borne and waterborne or foodborne diseases are anticipated to be the most impacted by climate change, given their sensitivity to these climatic factors. Climatic factors influence infectious diseases in a variety of ways, directly via impacts on pathogens, vectors, and transmission environment or indirectly by changing social behaviors or even disrupting disease control efforts [60]. In China, climate-sensitive infectious diseases including dengue fever (DF) [60–65], malaria [66,67], bacillary dysentery (BD) [68,69], hemorrhagic fever with renal syndrome (HFRS) [70], hand, foot, and mouth disease (HFMD) [71], and chikungunya [72] are projected to increase in case numbers and expand to nonepidemic areas under RCP scenarios (details in Supplementary Materials, p. 4–5). For example, one global study predicted that by 2080, the environmental suitability for DF occurrence in China is likely to expand to the north, exposing a larger population to the risk of DF compared to the year 2015 under 3 future scenarios (RCP6.0-SSP2, RCP4.5-SSP1, and RCP8.5-SSP3) [65]. For BD, one study including 316 Chinese cities projected that the cases attributed to future temperature would increase by >10% in some high-risk regions under different RCPs (2.6, 4.5, and 8.5) from 2030s to 2090s, especially under RCP8.5 [69].

### Study gaps

The available projection studies consistently indicated that limiting the temperature rise well below 2 °C would substantially mitigate the climate-related adverse health effects in China. However, there are questions remaining to be answered. First, limited projection studies have explored the disease burden due to nonoptimal temperature or other climatic factors under scenarios comparable to current Chinese carbon policies; instead, almost all studies have used the RCPs or SRES adopted by Intergovernmental Panel on Climate Change (IPCC). It is unclear if these scenarios are in line with the carbon-neutral policies in China, such as the coordinated governance of climate and air pollutants. Meanwhile, the local carbon-neutral scenarios of China and corresponding health effects specific to different carbon reduction pathways or sectors are still lacking. Current studies focused on urban regions and spatial disparities (urban versus rural) were seldomly studied. Second, the net changes for heat- and cold-related mortalities in the future have been most studied. However, the available evidence is often not comparable, given the variation across studies in area coverage, timescale, lag days, scenarios (temperature, demographics, and adaptation), definition of exposure (categorical or numerical) and outcomes (death count or years of life lost), as well as methods for constructing exposure–response relationships (Table S2). Third, for temperature-related morbidities, there have been limited projection studies in China, although there were some from developed countries. Fourth, whether transition to clean energy would reduce the adverse health effects of specific occupation types (e.g., coal workers) also needs to be resolved. Fifth, climatic infectious diseases have been projected under future RCPs, but impacts of other covariables such as vector control measures were seldomly considered.

## Health Effects of Extreme Weather Events Driven by Carbon Policies

### Flood

Health impacts of floods or high-volume precipitation onhealth have been well summarized in previous reviews [73]. In brief, floods/excess rain may pose immediate, early (<10 days), and long-term effects (>10 days) on human health, causing a range of flood-related morbidities and mortalities, including drowning, injuries, infections, zoonoses and vector-borne diseases, mental health, malnutrition, as well as poor management of chronic diseases due to difficulties with access to healthcare [73].

The effects of climate change, coupled with demographic changes, are anticipated to escalate the impact of flood disasters in the future [74]. An additional 0.5 °C increase in global temperatures from 1.5 to 2.0 °C is projected to double the increasing rate of duration and intensity of extreme precipitation in China in 2006–2100 compared with 1950–2005 [75]. Further, the IPCC Sixth Assessment Report estimated that by 2100, the global mean sea level will rise by 0.28 to 0.55 m and 0.63 to 1.01 m compared with 1995–2014 under SSP1-1.9 and SSP5-8.5, respectively [76], and could exacerbate the coastal floods. Two studies have projected the flood-related health impacts in China under RCPs, consistently projecting that burden of flood-related BD [77] and hepatitis A [78] would increase in China under RCP4.5.

### Drought, wildfire, and cyclone

Similar with flood, previous reviews and empirical evidence suggested that drought/low-volume precipitation, wildfire, and cyclones are also associated with a series of human diseases (Supplementary Materials, p. 6–7). Climate change is anticipated to exacerbate the severity of drought, wildfire/wildfire pollution, as well as cyclones in the future, even under RCP2.6 (Supplementary Materials, p. 6–7). However, there have been limited projection studies on their impact on human health in China. For drought, one recent study projected that annual average increased excess risk of dengue morbidity associated with Palmer drought severity index—a composite index reflecting temperature, precipitation, and evaporation—is expected to range from 15.03% under RCP2.6 to 60.77% under RCP8.5 by 2100. Pronounced increases occurred in areas characterized by mid-to-low latitudes, especially coastal areas [79]. For wildfire, the literature on its effect on mortality and morbidity burden under the context of climate change are mostly from the United States, which consistently estimated adverse health effects with certain variations by scenario, region, and health outcomes of interest (Supplementary Materials, p. 7) [80–83].

### Study gaps

The frequency and intensity of extreme weather events such as flood, drought, wildfire, and cyclone are expected to increase in China under the context of climate change, but there have been limited studies projecting their impacts on health in the coming decades. Empirical evidence suggested that flood and drought are related to increased risk of mortalities, exacerbation of chronic conditions, and mental health problems, but most projection studies have focused on the infectious diseases. Further, no projection studies were performed under scenarios comparable to current Chinese carbon policies.

## Co-Benefits of Air Quality Improvement Driven by Carbon Policies

The carbon policies in China would substantially improve the air quality in the coming decades (Supplementary Materials, p. 7–8). In this section, we summarized the published evidence on health effects of air pollutants (Supplementary Materials, p. 8–9) before we focus on the health effects attributed to air pollutant reductions driven by future carbon policies in China. Unlike climate change-related projections that exclusively used RCPs and SRES, a large quantity of studies took the carbon peaking and/or carbon neutrality into account when evaluating the air pollutant-related health effects, and we only focused on these in the review. Further, the widely used exposure–response function for mortality was on the long-term effect [84,85]; we therefore mainly focused on the long-term health co-benefits for mortality studies. By contrast, several morbidity studies tend to focus on short-term co-benefits [86].

### Health benefits of air quality improvement under future carbon policies

A large number of literature have quantified the health co-benefits attributed to specific pollutants such as PM_2.5_ and O_3_ under different Chinese carbon target policies in the future (see representative studies in Table S3). Health effects have been mainly on mortalities, although several studies have also evaluated benefits on morbidities. Further, the economic benefits from reducing air pollutants have also been estimated. Rationales for these projections were similar with those on nonoptimal temperature-related deaths. For exposure–response function, most studies have used 2 different modeling results—the global exposure mortality model (GEMM) [84] and integrated exposure–response (IER) model [85], which was partially responsible for the high degree of variance in co-benefit predictions. Similar to health effects of climate change driven by carbon policies, other uncertainties come from assumptions for policy scenarios, baseline incidence trend, as well as demographical changes (e.g., aging).

Achieving NDC of China and carbon neutrality can avoid hundreds of thousands of PM_2.5_-related mortalities per year compared with reference scenario without climate policy in China (Table S3). For example, achieving NDC in the electricity generation sector can avoid 19,900 premature deaths in 2030 and 368,600 in 2050 in China compared with the reference scenario without climate policy. Among the cause-specific mortalities considered, the most substantial health benefit is expected to arise from reduction in stroke cases—a category that was highly associated with PM_2.5_ exposure, followed by IHD, chronic obstructive pulmonary disease, and lung cancer [3]. The health effects vary by scenarios and geographical regions, and the reduction of deaths broadly follows the trend in population-weighted PM_2.5_ concentration changes [3,9,87–97], that is, more strict climate policies mean more air pollutant reductions and more health benefits. For example, carbon neutrality and other stricter climate policies such as Co-Benefit Energy with enhanced low-carbon policies, carbon peaking earlier than 2030, and 1.5 and 2 °C temperature targets could avoid 56,000 to 550,000 more PM_2.5_-related mortality than NDC in 2030 given that these stricter policies bring more reductions of PM_2.5_ concentrations [87,89,91,92,94,95,98,99]. Decarbonization pathway is also one important aspect of scenario. Zhang et al. [87] found that achieving carbon neutrality led by REs would bring about the most health benefits due to reductions in PM_2.5_ exposure, with a loss of life expectancy of 2.97 years per person in 2060. Carbon neutrality led by negative emission technologies (NEs) and NDC would win relatively less health benefits, with the loss of life expectancy of 0.78 and 1.34 years per person in 2060, respectively. For geographical variation, it is suggested that provinces with severe pollution and high population density (e.g., eastern China and the eastern areas of the southern and central China) would reap greater benefits from the PM_2.5_ reductions [3,87–89,91,94], whereas some areas of China such as northwest may experience a rise in PM_2.5_ concentration and therefore an increment of premature deaths [3]. Further, implementing region-specific policies that take into account energy and industry structure could enhance the improvement of PM_2.5_ air quality and amplify the associated health benefits [95,97,100]. For example, for heavy industry regions heavily relying on fossil fuel such as the Beijing–Tianjin–Hebei region, the implementation of stringent air pollution control policies along with ambitious earlier carbon-peaking measures leads to a decrease in PM_2.5_-related premature deaths by 1.8% in 2020–2030 [95]. By contrast, in Fenwei Plain where air pollutant emissions primarily originate from high-polluting industries and traditional use of coal for residential heating, the sole implementation of air pollution control policies could result in a reduction of PM_2.5_-related premature deaths by nearly 5% within the same timeframe [95].

Aging will exacerbate the PM_2.5_-related mortalities, given that older populations are more susceptible to these health effects [9,16,92,101]. With almost a quarter of the older people in the world, China would require substantial improvements in air quality to outpace this aging effect [102]. Studies suggested that only by combining the stringent clean air policy [e.g., ambitious pollution, maximum feasible reduction (MFR)] and the strict climate mitigation policies (e.g., well below 2 °C goal) would offset the impact of aging and get the PM_2.5_-related mortality decreasing in future decades (Table S3) [9,92].

O_3_-related mortalities will also show a declining trend under future carbon policies in China, although the reduction is smaller than PM_2.5_-related mortalities (Table S3) [89,93,96,98,103]. For example, Li et al. [93] found that under the 4% policy scenario (i.e., NDC), the number of premature deaths averted due to reduced O_3_ levels is 54,000 by 2030 compared with No policy scenario, nearly 60% of those from PM_2.5_. Similar with PM_2.5_-related health effects, areas heavily polluted by O_3_ could gain more health benefit from strategies aimed at carbon and pollution control, e.g., Beijing–Tianjin–Hebei region and central China [88].

Low carbon policies can also alleviate morbidities due to acute exposure to air pollutants (Table S3) [87,91]. Zhang et al. [87] identified that PM_2.5_-related bronchitis, hospitalization for CVD, emergency room visits, and hospitalization for respiratory disease under carbon neutrality scenarios could reduce by 47 to 71 million, 32 to 50 million, 3.1 to 4.65 billion, and 60 to 90 million, respectively, in 2060 compared with the reference scenario with no carbon emission constraints.

It is suggested that the benefits brought by Chinese carbon policies would be seen not only in China but also in boundary countries and even in overseas countries [90,93]. For example, in addition to 149,400 prevented PM_2.5_- and O_3_-related deaths from China’s 4% policy (i.e., NDC) in 2030, Li et al. [93] estimated that a total of 1,200, 3,500, and 1,900 premature deaths could be avoided in South Korea, Japan, and the United States, respectively.

In addition to the direct health effects, several studies have also evaluated the economic co-benefits attributed to Chinese carbon policies, which suggested that the monetized health benefits will surpass the costs of mitigation efforts [3,87,89,99]. For example, Cai et al. [3] found that 18 to 62% of the expenses incurred in fulfilling NDC in the electricity generation sector could be offset by the health benefits by 2030; the aggregate health benefits would grow substantially, reaching 3 to 9 times of the implementation costs by 2050.

### Study gaps

PM_2.5_-related health effects have been explored under both carbon peaking (i.e., NDC) and carbon neutrality polices, whereas O_3_-related health effects have less frequently been explored under NDC target. In addition, studies focusing on effects of other air pollutants such as NO_2_ under climate policies both inside and outside of China have been scarce. Given the relationship between NO_2_ concentrations and fossil fuel combustion, there are likely numerous health benefits that could be achieved through improved NO_2_ levels that have not been researched. Currently, mortality due to exposure to air pollutants under Chinese carbon policies was the main outcome (but again, these estimates were often not comparable because of large variations between studies), whereas the morbidity outcomes are still understudied. As the newest carbon target in China, pathways to carbon neutrality and the associated health effects require more research. For example, the potential health effects in population living/working near the main road, coal workers, and population relying on solid fuel heating under current Chinese carbon policies remain unclear. Many studies also do not always consider where the benefits from climate policies are occurring and from where the emission reductions are happening (e.g., how sources and receptors change across different climate policies), although quite a few studies have calculated [3,97] or compared [87] sector-specific emissions under climate and the corresponding health effects. Further, most studies projected air pollutant-related health effects independent of climatic factors and ignored their interactions (e.g., the dispersion and intensification of air pollutants under different meteorological conditions [104]). Finally, subnational studies focusing on local climate policies such as these [99,105] when evaluating health co-benefits should be conducted to provide region-specific reference for policy orientation.

## Other Health Impacts Related to Carbon Policies

In addition to shifts in climate and air quality, other mitigation strategies to achieve carbon peaking and carbon neutrality is also likely to yield positive impacts on public health. For example, the forest coverage and green space in cities would be markedly expanded to increase carbon sink [7]. In China, forest and green space have been linked to a series of health benefits including mental health, general health, and more favorable cardiometabolic and cerebrovascular outcomes [106]. In addition to public transport, the Chinese government encourages the advancement of sustainable transportation systems such as bicycle lanes and pedestrian zone [7], which indirectly increase the frequency and intensity of physical activities. Further, transition to green-carbon economy would also require shifting to sustainable and healthy diets. For example, decreased meat consumption can result in more substantial decreases in GHG emissions [107]. All the above beneficial changes under the new Chinese carbon neutrality remain to be studied. This review focused on the co-benefits. However, to have a full picture of the health impacts, emerging health threats linked to the new climate policies, such as novel chemicals, should also be considered and calculation of net health impacts is recommended.

## Policy Implications and Future Research Directions

Findings from the review have important policy implications. For example, a synergetic and stringent control of air pollution and GHG emissions at both the national and local levels is recommended to yield more health benefits, particularly in the context of an aging population. This could involve tightening air quality standards and enforcing them through legislation to drive fundamental improvements in air quality. In addition, prioritizing geographical regions with the greatest potential for health co-benefits, such as eastern China, is crucial for targeted policy-making and resource allocation. Moreover, the expansion of RE sources is suggested not only for their environmental benefits but also for the enhanced health outcomes they can bring. Technological optimization in key sectors should be encouraged to maximize these benefits. Finally, special attention should be given to vulnerable populations, including those with preexisting conditions (ischemic stroke, CVD, and IHD), as well as outdoor workers and the elderly, ensuring that they are adequately protected and considered in policy implementations.

In light of existing study gaps, several key areas are identified for future study. First, it is essential that future studies adopt a standardized modeling approach, given the considerable discrepancies observed in the magnitude of health impact assessments across current research. Second, health effects of distinct reduction policies tailored to different regions and sectors, as well as the effects with respect to morbidities under scenarios comparable to carbon peaking and carbon neutrality policies in China remain to be estimated. Third, beyond the commonly studied pollutants PM_2.5_ and O_3_, it is important to broaden the scope of research to include the effects of other air pollutants such as NO_2_ under current climate policies. Fourth, in addition to changes in climate and air quality, health effects of other mitigation strategies remain to be investigated. For instance, there is a need to address whether shifts toward clean energy sources might introduce new chemical substances into the environment and what health impacts they might have.

## Conclusions

Attainment of China’s carbon peaking and carbon neutrality targets involve economic and societal shifts that can greatly improve health by reducing GHG emissions and air pollution or promoting sustainable lifestyle. Modeling predicts a decrease in net temperature-related deaths and health benefits from improved air quality, including avoided PM_2.5_ and O_3_-related mortalities. This review provides up-to-date evidence of potential health co-benefits under Chinese carbon policies and highlighted the importance of considering these co-benefits into future climate policy making in China. Caution should be exercised when generalizing the findings to other nations endeavoring carbon reductions, taking into account different climatic, environmental, and socioeconomic contexts.

## Supplementary Materials

Supplementary 1 Supplementary Text Tables S1 to S3 References
