# Supplementary material for: Health Co-Benefits of Environmental Changes in the Context of Carbon Peaking and Carbon Neutrality in China
Source: Health Data Sci. 2024 Oct 2;4:0188. doi: 10.34133/hds.0188 (PMC11446102; doi:10.34133/hds.0188)
Supplement: Supplementary 1 — Supplementary Text Tables S1 to S3 References [file hds.0188.f1.pdf]

Supplementary material for

Health co-benefits of environmental changes in the context of carbon peaking and carbon neutrality in China

Feifei Zhang, Chao Yang, Fulin Wang, Pengfei Li, Luxia Zhang*

*zhanglx@bjmu.edu.cn

Search strategy and selection criteria

In this narrative review, we used a two-phase approach to identify and assess the health effects driven by Chinese carbon policies. In the first phase, we generated the outline of the Review by following three recent reviews which have comprehensively described the health effects of environmental changes due to climate change [1-3]. Related exposures include temperature, precipitation, extreme weather events (flood, drought, wildfire, and cyclone), as well as air pollution (**Figure**). In the second phase, we identified original research articles in English as of June 22, 2022 which quantitatively evaluated health effects in the future attributed to the aforementioned exposures through searches of PubMed, Web of Science and Google Scholar. We repeated the search twice, on 18 Jan 2024 and 10 June 2024, to include more recent studies. Primary terms included “carbon peak*” OR “carbon neutral*” OR “nationally determined contribution/NDC” OR “Paris Agreement”, combined with “China” and the individual exposure (i.e., temperature, precipitation, and flood etc.) in each round of search. We adjusted the search terms when the there is no eligible study for some certain exposures, e.g., i) we used the Representative Concentration Pathway (RCP) or Special Report on Emissions Scenarios (SERS) instead as the carbon emission scenarios (details shown in **Table**); ii) we removed the term “China” and looked at evidence from other countries for reference. Emphasis was placed on articles on health effects after 2015 when the NDC was initially proposed. We looked at earlier papers only when the available evidence was limited. Articles did not estimate health effects or did not consider carbon peaking or carbon neutrality policies or their equivalents, reviews, meta-analyses, and comments were excluded from the review. Data extracted included publication year, locations, climate policy scenarios, time horizons for projection, health outcomes of interest, methods for constructing exposure-response functions, and estimated health co-benefits.

Pathways of carbon peaking and carbon neutrality

Both government documents and simulated scenarios in published literature have summarised multiple pathways via which to attain carbon peaking in 2030 and carbon neutrality in 2060. On 22 September 2021, the State Council of China issued “Opinions on the Complete, Accurate and Comprehensive Implementation of the New Development Concept to Do a Good Job in Carbon Peaking and Carbon Neutrality” [4]. Further on 24 October 2021, the State Council of China issued the “Carbon Peaking Action Plan by 2030” [5]. These government documents formulated plans for

achieving goals of carbon peaking and carbon neutrality in China. In brief, achieving both goals is a multifaceted and systematic project concerning significant transformations across economic and societal structure. Main changes include optimising the industrial and energy mix, gradually phasing out the fossil fuels (e.g., coal), improving the energy efficiency, decreasing the energy consumption, and promoting green and low-carbon technologies (**Table 1S**) [4]. More detailed energy sector road map to carbon neutrality including the energy transition, sectoral pathways, technology and innovations as well as policy considerations have also been reported by the International Energy Agency (IEA) [6].

In addition to the government documents, peer-reviewed literatures have also explored pathways of carbon peaking and carbon neutrality. Main aspects include the CO_2 emission trajectories, energy restructuring and decarbonization [e.g., changes in carbon prices, total primary energy consumption (particularly coal), share of primary energy derived from fossil and non-fossil energy (e.g., solar, wind, nuclear, biomass), electrification ratio of the final energy consumption, low energy technology such as via enzyme catalysts etc.], application of negative emissions technologies [NETs, e.g., carbon capture and storage (CCS) technologies and direct air capture] , as well as the policy costs, by focusing on multiple sectors [7-9] [10-15] [16-18], or specific factors like electricity [19] and residential sectors [20]. Considering the complex interactions between climate change and air pollution, scenarios often take both into account. For example, under the scenario combining NDC and the current air pollution control policy, the proportion of China's primary energy sourced from coal is expected to gradually decline from 65% in 2015 to 48% by 2060; As a result, the annual anthropogenic CO_2 emissions are projected to reach a peak in 2030 at 12.4 Gt, followed by a reduction to 9.1 Gt in 2060 (**Table**) [8]. China's declaration of achieving carbon neutrality by 2060 aligns well with the 1.5 °C warming threshold or situates between the 1.5°C - and 2 °C-consistent climate targets [8,15]. The convergence of carbon neutral climate policies with the most stringent pollution control measures could potentially reduce the reliance on fossil fuels by 20% compared to the scenario set by the NDC goals scenario in 2015–2030, leading to a 15% decrease in CO_2 emissions [8]. By 2060, the annual anthropogenic CO_2 emissions are expected to plummet to 0.68 Gt, achieving net zero CO_2 emissions complemented by a natural carbon sink that absorbs ~0.7 Gt CO_2 annually (**Table**) [8].

Further, to be in line with the 1.5 °C-consistent target, China would need to reduce its total emissions of CH_4 and N_2O by over 70.6% and 52.2%, respectively, compared to the "no policy"

scenario [15]. CCS technologies such as conventional fossil fuels and bioenergy in conjunction with CCS technologies, are pivotal in reaching the 1.5 °C target, with captured carbon contributing to an average of 20% of the total reductions by 2050; there will be a necessity for renewables to expand by 175% on average relative to the no policy scenario, and the cumulative policy costs may represent 2.8 to 5.7% of the GDP by 2050 [15].

Given the vast geographical and socio-economic diversity within China, carbon emissions and intensities also show vast disparities across the country [21]. Socioeconomic factors play a crucial role in shaping the trajectories towards carbon peak and neutrality goals. For example, the increase in per capita GDP has been a primary driver of emission growth, particularly in the period before carbon peaking [21]. Furthermore, poverty eradication could inadvertently lead to increased carbon footprints and present a potential obstacle to carbon reduction initiatives. This is especially pronounced in less developed areas, where the growth rate of carbon emissions is expected to be as high as 4.0%, a figure significantly higher—by a factor of five—compared to more affluent regions [22].

Non-optimal temperature and morbidities

Empirical evidence has linked heat exposure to a series of morbidities including cardiovascular diseases (e.g., myocardial infarction hospitalization) [23,24], respiratory diseases [16], diabetes mellitus [25,26], kidney diseases (e.g., urolithiasis) [27-29], mental health and neurological diseases (e.g., mood disorders, schizophrenia, suicide, insufficient sleep, cognitive outcomes, and dementia- and/or Parkinson's disease- related hospital admissions) [30-37], as well as offspring and maternal health [e.g., preterm birth, stillbirth, low birth weight (LBW), psychiatric conditions, and gestational diabetes mellitus] [38-41], particularly under the prolonged heat exposure (i.e., heatwave). Less quantitative reviews have been on the cold-related morbidity; Available evidence suggests that cold exposure is likely to be positively associated specific cerebrovascular accident (e.g., intracerebral hemorrhages) [42], myocardial infarction hospitalization [24], respiratory morbidity in the elderly populations [16], diabetes mellitus [25,26], acute kidney injury in the elderly populations [43] , cognitive performance [44], as well as stillbirth [39]. As mortalities, these morbidities will also likely increase in the context of climate change. However, there are limited studies projecting the non-optimal temperature-related morbidities in China [45 46]. Here, we summarised the available evidence from other countries for reference.

Cardiovascular and/or cerebrovascular diseases morbidity is among the most studied outcome. For example, in Korea, it was projected that heat-related years lived in disability (YLD) for CVD and cerebrovascular diseases would increase from 0.86 YLD/1000 in 2011 to 1.11 in 2030 and 1.20 in 2050 under RCP4.5; Under RCP8.5 this would further increase to 1.25 YLD/1000 in 2030 and 2.47 in 2050 [47]. In Augsburg, Germany, it was projected that there would be a significant increase in myocardial infarction burden up to 2100 under RCP8.5, whereas limiting the warming below 2 °C (RCP2.6) would result in a negligible net change of myocardial infarction numbers [48]. By contrast, in Japan, net decreases in excess morbidity caused by temperature changes for cardiac arrest ouside the hospital were observed under higher emission scenarios (RCPs 4.5, 6.0, 8.5) in 2090–2099 compared with 2010–2019 [49]. Respiratory morbidity has also been projected. Under SRES scenarios A1B and A2, the total number of respiratory hospital admissions attributed to future temperature increases was predicted to see a twofold increase in Europe in 2021–2050 compared with the reference period [50]. Similar increasing trend for excess respiratory hospital admissions related to heat up to 2080–2099 has also been reported in New York State [51].

For kidney disease morbidity, two studies from the US projected that the heat-related nephrolithiasis will increase under SRES A1B [52] and RCPs (4.5, 8.5) [53]. The study by Brikowski, et al. in 2008 estimated that high-risk areas for nephrolithiasis in the US would expand to north, with the proportion of population from high-risk areas increasing from 40% in 2000 to 56% in 2050 and to 70% by 2095 under SRES A1B [52]. One further study in 2022 estimated that total statewide nephrolithiasis occurrences attributable to heat in South Carolina were projected to increase by 2.2% under RCP4.5 and by 3.9% under RCP 8.5 by 2085–2089 when compared to the 2010–2014 [53].

There have been limited studies projecting future mental health- and neurological disease-related mortalities in China. One study in China has estimated the effects of temperature on high-stakes cognitive performance using data of the National College Entrance Examination under four RCPs (2.6, 4.5, 6.0, and 8.5) and found that western counties are projected to see a larger drop in examination scores (1.8%–1.9%), potentially exacerbating the inequalities under climate change [54]. Further, one study on sleep in the US found that climate change could potentially lead to around six extra nights of insufficient sleep per 100 individuals by 2050 and 14 nights under RCP8.5 by 2099 compared to 1981–2010 [35]. For neurological disease, one study has projected

that heat-related admissions for dementia are expected to rise by 263% and 294% under RCP2.6 and RCP8.5 by 2040 compared to baseline levels in 2010 in England [36].

Several studies have also explored future temperature-related effects on offspring and maternal health outside of China. In the US, under A2 scenario based on the National Center for Atmospheric Research Community Climate System Model 3, it was projected that mean birth weights would decline by 7.5 g on average among whites and by 11.5 g for blacks in 2070–2099; For low birth weight (LBW), there would be 5.9% and 5.0% increase in the probability for whites and blacks, repectively[55]. A similar average net reductions of 4.6 g in birth weight in 2070–2099 under RCP8.5 were reported in New York city by Ngo et al, though a positive net impact was reported under RCP4.5 scenario, which favors the adverse effect of high carbon emission scenarios [56]. Similarly in Hungary, it is projected that the mean birth weight is possibly to decline by 15.3 g, and the prevalence of LBW is likely to increase by 0.19% under RCP8.5 in 2040−2059 [57].

Climate-sensitive infectious diseases

Predicting the climate change on DF has been challenging, involving modeling future climate change (climate model), how dengue fever incidence changes with climate (DF model) and how climate changes will affect mosquitoes' vectorial capacity (mosquito model) [58-63]. Based on future weather data variables (temperature, relative humidity, and precipitation), one study projected that the incidence of DF in Guangzhou in 2020–2070 is distinct under the different RCPs (2.6, 4.5, 6.0, and 8.5), particularly during summer and fall; Higher peaks and overall incidence of DF cases are anticipated to occur under RCP8.5 [58]. One global study predicted that the environmental suitability for DF occurrence in China are likely to expand to north, and a greater number of individuals will be susceptible to DF in 2080 when compared to 2015 under three future scenarios (RCP6.0-SSP2, RCP4.5-SSP1 and RCP8.5-SSP3); The future DF cases vary by the RCP/SSP scenarios, with greater number of cases under RCP8.5-SSP3, followed by RCP6.0-SSP2 and RCP4.5-SSP1 [63]. Moreover, studies have also predicted DF distribution by projections of mosquito population and distribution from mosquito model as well as climate model [59-61]. For instance, one study projected that areas prone to high risk of DF in China would expand under all RCP scenarios (2.6, 4.5, 6.0, 8.5) in 2020s–2100s compared to the baseline,

especially under RCP8.5; By 2100s under the RCP8.5 scenario, the individuals and expanded high risk areas would increase by 4.2 times and 2.9 time compared to the baseline [60].

Similarly, two types of studies have been performed to project the future malaria incidence in China. The first type projected malaria incidence based on the weather–malaria relationship [64,65]. For example, one study projected that future malaria related to weather in China would increase by 5.5%–49.8% and 6.9%–79.6% up to 2100 compared to the baseline in 1985–2014 under RCP4.5 and RCP8.5, respectively [65]. By contrast, the second type has assessed the impact of climate change on regions suitable for distribution of malaria vectors. Using maximum entropy species distribution model, Ren et al projected that the impact of future changes in climate and land use would likely increase the distribution of the four main malaria vectors (*An. dirus*, *An. minimus*, *An. lesteri* and *An. sinensis*) in future decades under RCPs (2.6, 4.5 and 8.5) compared with the current environmentally suitable area, except for *An. dirus* and *An. minimus* under RCP2.6; The individuals exposed to the four main malaria vectors would also show a significant net increase in the 2030 s and 2050s [66].

Three studies projected an upward trend of BD incidence under climate change scenarios in China [67-69]. For example, one study including 316 Chinese Cities projected that temperature-attributed BD cases would increase in most regions of China under different RCPs (2.6, 4.5, 8.5) from 2030s to 2090s, especially under RCP8.5; The regions of Northern, Northeast, Inner Mongolia, Northwest, and Southern China have been pinpointed as zones with heightened vulnerability to future outbreaks of BD, with estimated increases above 10% compared with baseline by the 2090s under RCP 8.5; When factoring in adaptation measures—such as a 30% reduction in the exposure–response function between temperature and BD)— as well as considering the projected population changes (based on a medium fertility variant and maintaining the current age distribution as forecasted by United Nations Population Estimates and Projections), the projected percentage differences in BD cases due to projected temperatures relative to baseline values were reduced but still remained positive for most regions except Tibet [69].

Other infectious diseases under the context of climate change have also been projected in China, including hemorrhagic fever with renal syndrome (HFRS) [70], schistosomiasis [71], hand, foot, and mouth disease (HFMD) [72], and Chikungunya [73], suggesting an increasing trend incidence or

expansion of transmission-suitable areas (except that HFMD may decline with climate change in temperate zones of central and eastern China [72]).

Drought and human health

Drought can cause agricultural production loss and food insecurity, a lack of freshwater, increase drought-associated heat events and wildfires, and alter vector-borne disease transmission dynamics (e.g., mosquito densities increases due to domestic water storage tanks as a way to cope with water shortages drought events) [74]. Droughts have been linked to a series of health problems including malnutrition and mortality, water-related disease caused by microbial contamination and cyanobacteria blooms (e.g., E.coli O157, leptospirosis), skin, eye and louse-borne diseases (e.g., conjunctivitis), airborne and dust-related conditions such as coccidioidomycosis, vector-borne disease, mental disorders and aggravating chronic diseases [74].

China was among the top one/two countries with both the largest number of affected population and drought-related mortalities in 1900–2012 [74]. China is projected to face an increase in both the frequency and intensity of droughts with longer durations in more areas in future decades, particularly under higher GHG emission scenarios [75]. There have been limited studies projecting the health impacts related to the future drought events in China[76]. One study in Africa projected that drought-related cholera outbreaks are likely to be offset by improved socio-economic conditions under a combination of drought, temperature (RCP4.5, 6.0 and 8.5), poverty and freshwater withdrawal scenarios [77]. Further studies are required to comprehend the effects of drought on human health in China.

Wildfire and human health

Wildfire interplays with both climate change and air quality—Changes climatic conditions including temperature, rainfall, and wind speed can impact wildfire activity, and wildfire smoke generate adverse air pollutants such as particulate matter, carbon monoxide, nitrogen oxides, and volatile organic compounds [78]. Climate change is projected to amplify the intensity of wildfire/wildfire-related pollution in the future. For instance, Wu et al found that climatic factors increase wildfire frequency in southern China and north-eastern China under both RCP2.6 and RCP8.5 in 2050 and 2070 [79].

There has been a scarcity of studies concentrating on the impact of wildfires on people's health. In addition to direct injuries and direct fatalities, wildfire cause indirect health problems related to smoke/Fine particulate matter ($PM_{2.5}$) exposure including premature mortality, hospitalisation and visits to emergency room due to respiratory illness (e.g., asthma, COPD) and cardiovascular illness, psychological disorders, and adverse birth outcomes [78]. In China, there have been a scarcity of studies concentrating on the effect of wildfires on human health, but several global studies including China reported the adverse health effects of wildfires [80,81]. Research concerning the potential mortality and morbidity burden linked to air pollution from future wildfire smoke/PM exposure in the context of climate change are mostly from the US, which have consistently estimated adverse health consequences with some heterogeneity by scenario, region, and health outcomes of interest [82-85]. For example, it is projected that in western US, the joint influence of wildfire-related $PM_{2.5}$ exposure changes and population projections would result in an excess burden of premature mortalities in the 2090, with an 2.6 times (RCP4.5) or 3.5 times (RCP8.5) increase compared with the baseline period of 1996–2005 [84]. The smoke-related asthma events (emergency room visits and hospitalizations) are anticipated to rise at a rate of 15.1 visits per 10 000 individuals in the Western US in the 2050s than baseline years of 2003–2010 under RCP8.5 and Integrated Climate and Land-Use Scenarios ICLUS A2 population scenario [85].

Cyclone and human health

Tropical cyclone (or typhoon/hurricane) is a destructive extreme weather often occur together with strong wind, heavy storm, and flooding. Similar with flood, previous reviews suggested that cyclones are associated with a series of diseases including direct mortalities and injuries and indirect effects including infectious diseases, respiratory illness, exacerbation of pre-existing chronic conditions, and mental disorders [86-88]. South-eastern China is frequently hit by cyclones every year, especially in Guangdong, Fujian, Hainan and Zhejiang Province; it is estimated that 490 cyclones landed in China in 1951–2017, with an average of 7.4 cyclones per year [89]. It is projected that the intensity of tropical cyclones will increase due to the climate change, particularly under RCP8.5 [90]. Moreover, the climate change, together with urbanization will exacerbate the cyclone flood hazard in urban areas such as Guangzhou Metropolitan Area [91]. Empirical evidence from China suggested that tropical cyclones are related to increased risk of a couple of infectious diseases including infectious diarrhea, hand, foot and mouth disease, and

dengue fever [89,92-95]. However, there have been limited studies projecting the health impacts of future cyclones.

Air pollution and carbon policies

GHGs and air pollutants share emission sources, mostly from burning of fossil fuels. Therefore, climate policies targeting GHGs also reduce emissions of air pollutants [96]. For example, quite a few simulation studies suggested a lower concentration of multiple air pollutants (PM, O_3, NO_x, and SO_x etc.) under future climate policies including carbon peaking and carbon neutrality in China [8,9,14,97,98]. Xing et al found that except a reduction of 38% CO_2, achieving the mitigation goals set out in the INDC also provides the added benefit of reducing emissions of black carbon (BC), $PM_{2.5}$, and SO_2, with reductions of 21%, 16%, and 31% in 2030 from the residential sector compared with the reference scenarios with no intervention of efficient technology [20]. With combination of NDC and current air pollution control policies, Cheng et al found that China is on track to meet its annual air quality standard for $PM_{2.5}$ (35 μg/m^3) by 2030; Achieving China's carbon neutrality targets could further reduce the $PM_{2.5}$ concentration, with 78% of Chinese population (or 85% of cities) below the WHO guideline (10 μg/m^3) by 2060 [8]. Further, Shi et al have assessed the changes of multiple air pollutants under the carbon neutrality target; Compared to the year of 2019, emissions of precursors of $PM_{2.5}$ and O_3 including SO_2, NO_x, primary $PM_{2.5}$, and volatile organic compounds expected to see a significant reduction by 28% to 44%, and in 2030 and by 61% to 93% in 2060 respectively; national annual average concentrations of $PM_{2.5}$ is expected to be 27 μg/m^3 and the 90th percentile of daily maximum 8-h O_3 concentrations will be 129 and 93 μg/m^3 in 2030 and 2060, respectively [14].

Air pollution and human health

Air pollution ranks as one of the top five contributors to deaths and disease burden in the globe [99]. Air pollution could affect multiple systems, including cardiovascular, respiratory, endocrine, urinary, gastrointestinal, immune, neurologic and psychiatric systems [100,101]. A mapping review of published systematic reviews and meta-analyses discussed the adverse effect of air pollution on health and showed that exposure to air pollutants (e.g., $PM_{2.5}$, PM_{10} and NO_2) is particularly related to higher risks of mortality and cardiopulmonary outcomes (e.g., asthma, COPD, and stroke) [102]. Air pollution from specific sources including traffic [103], wildfires [78], indoors (e.g., coal and biomass Fuels [104]) have also been explored, consistently showing an adverse health

effect. Similar with temperature, air pollution affect worker's health and productivity; For example, exposure to particulate matter in the workplace is linked to an increased risk for cardiovascular disease [105] and reduced productivity [106]. Further, studies have retrospectively assessed the effectiveness of interventions (e.g., Hong Kong's 1990 low-sulphur fuel law, air pollution reduction during the 2008 Beijing Olympic Games, and China's Air Pollution Prevention and Control Action Plan) to reduce pollutant concentrations in improving associated health outcomes [107] [108,109], and reveal the possible causal relationships between air pollution and health problems.

Supplementary tables

Table 1S Phased goals of carbon peaking and carbon neutrality set by Chinese government

Time scale	Energy consumption per unit of GDP	CO_2 emissions per unit of GDP	Proportion of non-fossil energy consumption	Forest coverage rate and forest stock volume
By 2025 (end of “14th Five-Year Plan” period)	Reduce by 13.5% compared to 2020.	Reduce by 18% compared to 2020.	Reach about 20%.	Reach 24.1% and 18 billion cubic meters, respectively.
By 2030 (end of “15th Five-Year Plan” period)	Reduce significantly.	Reduce by more than 65% compared to 2005. (CO_2 emissions will reach the peak and then decline steadily).	Reach about 25%. (The total installed capacity of wind power and solar power is projected to exceed 1.2 billion kilowatts).	Reach about 25% and 19 billion cubic meters, respectively.
By 2060	The energy use efficiency will reach the international level.	Carbon neutrality will be achieved.	Reach over 80%.	–

Table 2S Projected non-optimal temperature-related deaths in China

Study	Locations	Scenarios	Time horizon	Health outcomes	Exposure-response (E-R) relationship: methods and values	Main findings
1. Heat-related deaths in China						
Liu et al., 2023 [110]	Seven regions of China including South, North, East, Central, Northeast, Southwest, and Northwest	Combination of future temperature and population scenarios: SSP1-2.6, SSP2-4.5, SSP3-7.0, and SSP5-8.5	2010 vs. 2010-2100	Heatwave-related mortality	Exposure-response functions derived from previous studies	1. Under the SSP5-8.5 scenario, the annual tally of excess deaths attributed to heat is expected to increase from 100,500 in 2010 to 1,926,400 in 2081–2100, driven by the interplay of climate change, population expansion, and aging.<br>2. Burden of deaths: SSP5-8.5> SSP3-7.0> SSP2-4.5> SSP1-2.6, Central and south-east coastal regions>other regions.<br>3. By the year 2100, the effect of aging would outweigh that of climate change and population expansion under all four scenarios in 2010-2100.<br>4. The economic costs of excess Years of Life Lost (YLL) due to heat would range from 43.7 to 129.8 billion dollars (in 2005 dollars) in 2100 under different SSP scenarios: SSP1-2.6, SSP2-4.5, SSP3-7.0 and SSP5-8.5, which would be approximately 0.09%–0.28% of China’s GDP.
He et al., 2022 [111]	Three east Asian countries including China	1. Future temperature scenarios: SSP1-RCP2.6, SSP2-RCP4.5<br>2. Future adaptation: the threshold for a hot night was established based on 95th percentile of expected temperature in the future, rather than using the constant threshold derived from baseline temperature	2010s vs. 2010s-2090s	Hot night-related mortality	1. Two-stage analysis: city-specific E-R relationship using the distributed lag non-linear model (DLNM) with a quasi-Poisson regression in 1981–2010 plus univariate meta-analysis to pool the E-R relationship.<br>2. The relative risk at the 99th percentile of hot nights ranges from 1.3 to 1.5.	1.The mortality burden is more significant in North China compared to in South China.<br>2. In South China, the proportion of mortality attributable to hot nights exceeds the proportion of mortality attributable to daily mean temperature even under SSP1-RCP2.6 after 2070s. Conversely, in North China, the proportion of mortality attributable to daily mean temperature consistently surpasses that linked to hot nights.

		distribution.				
Chen et al., 2022 [112]	31 provinces in mainland China	1. Future temperature scenarios: RCPs (2.6, 4.5, 8.5), including 1.5°C climate goal related to achieving carbon neutrality by 2050s by using the temperature in 2021−2040 under the RCP2.6<br>2. Population scenario: SSP 2	1986-2005 vs. 2021-2040, 2051-2070, 2081-2100 (20-year averaged)	Heatwave-related mortality	Two-stage analysis: location-specific E-R functions using a Poisson generalized linear model in 2007-2013 plus a meta-analysis to pooled effects of heatwave on mortality in 31 provinces from seven climate zones.<br>Confounders: air pollution index and relative humidity.	1. Under RCP8.5, the mortality is expected to keep climbing, while under RCP2.6 and RCP4.5 scenarios, it is expected to continue to rise until 2040−2060 and then decline thereafter. Even under RCP2.6 scenario, the number of deaths is expected to increase to 31,278 deaths by mid-century, marking a 205% increase from the baseline period. By 2090, the number of deaths due to heatwaves is expected to increase by 1.9 to 6.7 times under three RCP scenarios.<br>2. Burden of deaths: RCP8.5>RCP4.5>RCP2.6>1.5 °C,<br>East and central China > other regions.<br>3. Climate change is identified as the primary factor driving the increase in attributable deaths in the foreseeable future up to 2060, accounting for 78% of the overall change.
He et al., 2021 [113]	364 locations	Future temperature scenarios: RCPs (2.6, 4.5, 8.5)	1990s vs. 2030s, 2060s, 2090s (decadal)	Compound hot extremes (CHE, a hot night with a following hot day)-related all-cause mortality	1. Two-stage analysis: location-specific E-R relationship using the DLNM with a quasi-Poisson regression in 2006–2017 plus multivariate meta-analysis to pool the E-R relationship. Confounders: year for long-term trends, relative humidity, day of week, and PM_{10}.<br>2. Cumulative relative risk (RR) of CHE (as a binary outcome) over 0–7 days lag: 1.23 (1.19,1.28).	1. Without climate change adaptation and population change, deaths attributable to CHEs increase by 7–19 times in 2090s under RCP4.5 and RCP8.5 compared to deaths during the baseline period. For RCP2.6, CHE-related-death increase by 3 times in the 2090s.<br>2. Burden of deaths: RCP8.5>RCP4.5>RCP2.6.
Yang et al., 2021 [114]	161 surveillance points (64 districts and 97 counties, covering 73 million people)	1. Future temperature scenarios: RCPs (4.5, 8.5)<br>2. Population scenarios: SSPs (1–5)	2010s vs. 2030s, 2050s, 2090s	Heat-related all-cause and cause-specific mortalities	1. Two-stage analysis: location-specific E-R relationship using the DLNM with a quasi-Poisson regression in 2007–2013 plus multivariate meta-analysis to pool the E-R relationship. Confounders: main model: year for seasonality and the time trend, day of week; sensitivity analysis model: daily mean relative humidity or mean wind speed or $PM_{2.5}$ and O_3.<br>2. Cumulative RR of daily mean	1. Assuming there is no adaptation or population shifts, decadal heat-related excess deaths increased by 1.2–2.9 times under two RCPs.<br>2. Burden of deaths: Population ageing>no population change,<br>People with cardiorespiratory diseases>with other causes,<br>The elderly>the youngsters,<br>Females>males,<br>Southern, eastern, central, and northern

					temperature for all-cause mortality over 0–14 days lag: U- or reverse J-shaped; RR of high temperature relative to the minimum mortality temperature (MMT) was highest at 1.87 at 36°C nationally.	China > other regions in China,<br>Low educational attainment>high educational attainment.
Wang et al., 2019 [115]	27 metropolises (account for 18.6 and 29.7% of the national total pop and GDP, respectively)	1. Future temperature scenarios: RCPs (2.6, 4.5)<br>2. Scenarios of the population by age and gender, and the GDP: SSPs (1–5)	1986–2005 vs. 2060–2099	Heat-related all-cause mortality	1. Location-specific E-R relationship using the DLNM in 2007–2013. Confounders: year for long-term trends, day of week, and public holidays.<br>2. Cumulative RR of daily maximum temperature over 0–10 days lag: U-, V- or J-shaped and vary by age and sex; RR of high temperature relative to the MMT was highest at 2, e.g., in female non- working age population	1. With adaptation: annual heat-related mortality per million pop increase by 1.8–2.2 times under various RCPs and SSPs.<br>2. Burden of deaths: RCP4.5>RCP2.6,<br>Scenario of adaptation<No adaptation,<br>Male>Female,<br>Non-working age>Working age.
Liu et al., 2019 [116]	Guangzhou	1. Future temperature scenarios: RCPs (2.6, 4.5, 8.5)<br>2. Scenarios of population expansion and ageing (no population change, and low, medium, and high population expansion)<br>3. Adaptation scenarios (no adaptation and three scenarios of increased adaptation to heat—S1 scenario (increase by 8.92%/ decade), S2 scenario (increase by 4.6%/ decade), and S3 scenario (MMT increase by about 0.2 °C/decade)	1980s vs. 2030s, 2060s, and 2090s,	Heat-related all-cause YLLs	1. E-R relationship using the DLNM with Gaussian distribution in 2010–2015. Confounders: year for secular trends, day of week, relative humidity, wind speed, NO_2, and PM_{10}.<br>2. Relationship between daily mean temperature and YLLs along lag 0−1 days: U-shaped; YLLs of high temperature relative to the minimum YLL temperature was highest at around 390 among the total population.	1. There is a significant increase in *YLLs* related to heat in the 2030s, 2060s, and 2090s.<br>2. Burden of deaths: RCP8.5>RCP4.5>RCP2.6,<br>Scenario of rapid population expansion and ageing>no population change,<br>Scenario of adaptation<No adaptation (75% and 20% *YLLs* for the total population and the elderly will be counteracted by adaptation, respectively).
Guo et al., 2018 [117]	15 Chinese communitie s (Global studies including China)	1. Future temperature scenarios: RCPs (2.6, 4.5, 6.0, 8.5)<br>2. Three scenarios of population change (high variant, median	1971–2020 vs. 2031–2080	Heat-related all-cause mortality	1. Two-stage approach: community-specific E-R relationship based on a time series Poisson regression model in 1996–2008 plus meta-regression to pool the overall cumulative risk ratios. Confounders: seasonality, long-term	1. Without adaptation, mean percent change of excess deaths attributed to heatwave between years 2031to 2080 and years 1971 to 2020 ranges 84%–308%.<br>2. With full adaptation (hypothetical adaptation to the 95th percentile

		variant, and low variant).<br>3. Adaptation scenarios (no adaptation and hypothetical adaptation)			trend, day of the week.<br>2. Overall cumulative OR of heatwaves over lag 0–10 days varied by community, ranging 1.02-1.2 in China.	temperature), mean percent change of excess deaths attributed to heatwave ranges 1%–35%.
Zhang et al., 2018 [118]	Three districts: PuKou (PK) district of Nanjing city, WanZhou (WZ) district of Chongqing city, and YueXiu (YX) district of Guangzhou city	Future temperature scenarios: RCPs (2.6, 4.5, 6.0, 8.5)	1971–2015 vs. 2051–2095	Heat-related all-cause mortality	1. Region-specific E-R relationship using the DLNM combined with a linear regression in 2007–2012. Confounders: for main model: mean temperature, humidity, long-term and seasonal trends, day of the week, and holidays; for sensitivity analysis: maximum temperature, minimum temperature, and air pollution index metric.<br>2. Crude mortality rate over 0–3 days lag increased with Heat Wave Intensity Index (HWII) percentiles and varied by regions.	1. The predicted average annual loss (AAL) of life in three districts under four RCPs are approximately 5~161 times of the AAL during the baseline.<br>2. Burden of deaths: WZ Chongqing>PK Nanjing>YX Guangzhou,<br>Elders (Aged 65+)>the youngsters (Aged 0–64)
Li et al., 2018 [119]	51 largest Chinese cities (one third of the total population in China)	1. Future temperature scenarios: RCPs (2.6, 4.5, 8.5)<br>2. Population projection scenario [no population change, and population growth rates from United Nations' 2015 RWPP (Revision of World Population Prospects)]<br>3. Adaptation scenarios (no adaptation and adaptation using the "analogue city" approach)	1970–2000 vs. 2041–2060, 2061–2080	Heat related all-cause mortality	1. City-specific temperature-mortality risk estimates by a extensive review of published literature in China.<br>2. Beta value for all-cause mortality for each 1 °C increase is estimated to range 0.005–0.076.	1. Excess annual heat-related mortality increases by 1%–3% compared to the baseline under three RCPs.<br>2. Burden of deaths: RCP8.5>RCP2.6, Scenario of adaptation<No adaptation, Cities in the North, East and Central regions of China>other regions of China.
Chen et al., 2017	104 counties of Jiangsu	1. Future temperature scenarios: RCPs (4.5,	1981–2005 vs.	Heat-related all-cause and cause-	1. County-specific E-R relationship using the DLNM with a quasi-Poisson	1. The mortality linked to heat for total non-accidental causes, cardiovascular diseases,

[120]	Province	8.5)<br>2. Population projection scenario [no population change and SSPs (1–5)]	2016–2040, 2041–2065	specific mortalities	regression in 2009–2013 plus a Bayesian hierarchical model to pool the county-specific estimates.<br>Confounders: year for long-term trends, day of week.<br>2. Cumulative RR of daily mean temperature for all-cause mortality risk in 0–6 days lag comparing the 99th with 75th percentiles of temperature: 1.43 in non-urban counties and 1.26 in urban counties.	respiratory conditions, stroke, ischemic heart disease (IHD), and chronic obstructive pulmonary disease (COPD) is elevated when compared to the baseline figures. Variations in population size change exerted only a modest influence<br>2. Burden of deaths: May to September>other months, RCP8.5>RCP4.5,<br>Non-urban residents> urban residents.
Li et al., 2016 [121]	Beijing	1. Future temperature scenarios: RCPs (4.5, 8.5)<br>2. Population projection scenario [“no change” scenario and “aging” scenario (i.e., low, medium, and high variant scenarios of population growth developed by the United Nations)]<br>3. Adaption to heat scenarios (increase 5%, 15%, and 30%, and 50% adaptation)	1980s vs. 2020s, 2050s, and 2080s	Heat-related all-cause mortality among adults 65 years and older	1. E-R relationship using the DLNM with a Poisson regression in 2008–2011.<br>Confounders: year for seasonal and long-term trends, day of week.<br>2. Cumulative RR of daily mean temperature in 0–14 days lag: U-shaped with the MMT at 21.4 °C, RR of high temperature relative to the MMT was highest at 2.4.	1. Without adaptation, heat-related deaths increased by 38%–207% compared to the baseline under both RCPs.<br>2. Burden of deaths: RCP8.5>RCP4.5, Scenario of population growth>No population change,<br>Scenario of adaptation<No adaptation.
**2. Net temperature-related deaths**						
Wang et al., 2022 [122]	Hong Kong	1. Future temperature scenarios: RCPs (2.6, 4.5, 6.0, 8.5)<br>2. Three different adaptation scenarios [With 0.25 °C increase in threshold within each two decades; With 5% slope reduction in exposure-response function (ERF) every 20 years; combined absolute threshold	2014–2018 vs. 2030s, 2050s, 2070s, and 2090s	Heat- and cold-related all-cause and cause-specific moralities	1. The yearly age-standardized mortality rates (ASMRs) in 1976–2018 were linked to annual hot and cold degree-days using quasi-Poisson generalized additive model.<br>Confounders: year of the study.<br>2. For all-cause mortality, beta value for the association with hot and cold temperatures were 0.00233 and 0.00019, respectively.	1. Generally, yearly ASMRs are expected to show a net increases under the RCP scenarios 4.5, 6.0 and 8.0, primarily due to the rise in mortality from excess hot days outpacing the reductions from excess cold days. Under RCP2.6 scenario, the ASMR was estimated to decline after 2050s and became negative after 2070s.<br>2. Burden of deaths: RCP8.5>RCP4.5/RCP6.0>RCP2.6,<br>Aged over 75 years> aged 74 and below, Cardiovascular deaths> respiratory deaths, Scenario of adaptation<No adaptation.

		shift of 0.25 °C and 5% reduction in slope)				
Sun et al., 2021 [123]	105 counties of China	Three scenarios—low, medium, and high-speed scenarios—were formulated by incorporating effect-modifiers and leveraging the correlation between SSPs and the RCPs for future temperature, birth rate, mortality, and gross GDP	2013–2017 vs. 2050s, 2080s	Heat- and cold-related all-cause moralities	1. E-R relationship in 2013–2017 through DLNM and a meta-regression.<br>2. Cumulative RR over 0–14 days of lag: U-shaped with the highest RR at around 1.75 and 1.6 for cold and hot temperatures, respectively.	1. The projected number of temperature-related excess deaths is expected to rise in the 2050s and decline in the 2080s.<br>2. The temperature-related excess mortality is projected to increase in the 2050s and decline in the 2080s in the East, the North, and the Central, or keep declining in the Northeast, the Northwest, and the Southwest. Most regions are expected to see a decrease in the temperature-related excess mortality at last, with the exception of South China, where an upward trend is observed.<br>3. Burden of deaths: Medium-speed scenario<low and high-speed scenario
Zhu et al., 2021 [124]	272 Chinese cities in 31 provincial administrative regions in China, excluding Hong Kong, Macao, and Taiwan	1. Future temperature scenarios: RCPs (4.5, 8.5)<br>2. Population scenarios: SSPs (1–5)	2010 vs. 2050	Heat- and cold-related all-cause moralities	1. City-specific E-R relationship from a previously published study [125] which used overdispersed generalized linear model (GLM) in combination with a DLNM s in 2013–15 plus multivariate meta-regression analysis to pool the effect estimates by national and regional levels.<br>Confounders: year to control for seasonality and long-term trends, relative humidity, and day of the week.<br>2. Cumulative RR of daily mean temperature over ≤21 lag days: Inversely J shaped; RR was highest at around 2.45 and 1.35 for extreme low and high temperature, respectively.	1. In most cases, the temperature-related deaths are projected to drop by 1.6%–9%, suggesting that the increase in heat-related deaths caused by climate change will be counterbalanced by the reduction in cold-related deaths. However, under the most populous scenario (SSP3), the temperature-related deaths will rise by 3%.<br>2. Burden of deaths: populous scenario > non-populous scenario,<br>RCP8.5>RCP4.5,<br>Urban heat island (UHI) > other areas.
Gu et al., 2020 [126]	Ningbo City	Future temperature scenarios: RCPs (4.5, 8.5)	2010s vs. 2030, 2050s, 2090 s	Heat- and cold-related all-cause and cause-specific moralities	1. E-R relationship using the DLNM with a quasi-Poisson regression in 2009–2018.<br>Confounders: for main model: year to control for seasonality and long-term trends, relative humidity, and day of the week. For sensitivity analysis: $PM_{2.5}$, O_3.<br>2. Cumulative RR of daily average temperature over 0–21days lag:	1. Net temperature related total mortality fraction are projected to change by -0.54% (95% eCI: -1.69% to 0.71%) and -0.38% (95% eCI: -2.73% to 2.12%) in 2090s under RCP4.5 and RCP8.5, respectively.<br>2. Among 15 causes, a net decrease in the future temperature-related mortality burden was noted for 10, with estimates ranging from -5.02% in mental disorders to -1.01% in chronic lower respiratory disease.

					Inversely J shaped for total mortalities; RR was highest at around 1.9 and 1.4 for extreme low and high temperature, respectively.	Conversely, the remaining diseases are anticipated to see a possible net increase in temperature-related death burden, with estimates ranging from 0.44% in ischemic heart disease and 4.80% in external causes.
Dong et al., 2020 [127]	Yancheng	1. Future temperature scenarios: RCPs (4.5, 8.5)<br>2. Population scenarios [a constant scenario and two SSPs scenarios (2, 5)]<br>3. Adaptation scenario (MMT will rise at 0.2°C /decade)	2011–2020 vs. 2030s, 2050s 2070s	Heat- and cold-related respiratory disease moralities	1. E-R relationship using the DLNM with a quasi-Poisson regression in 2014–2017.<br>Confounders: year and calendar month to control for seasonality and long-term trends, day of the week, relative humidity, wind speed, atmospheric pressure, O_3, NO_2, PM_{10}, and SO_2.<br>2. RR of daily mean temperature at 21-day lag: U shaped; RR was highest at around 5.1 and 3 for extreme low and high temperature, respectively, and MMT is 20.5°C.	1. Assuming no adaptation, under SSP population scenarios, the reduction in cold-related deaths is expected to counterbalance the increase in heat-related deaths, resulting in a net decrease in temperature-related mortality in the 2050s and 2070s.<br>2. Burden of deaths: Future changes in population are projected to have a greater impact on respiratory mortality compared to impact from future climate change, Scenario of adaptation<No adaptation
Yu et al., 2019 [128]	158 counties across China	Future temperature scenarios: RCPs (2.6, 4.5, 6.0, 8.5)	2004–2012 vs. 2041–2060, 2061–2080	Heat- and cold-related moralities	1. Modelling the log of the annual mortality rate to the number of days when the daily mean temperature is in the jth of the 10°F bins over 2004 to 2012.<br>Confounders: humidity, precipitation, age structure of the population, county, and year.<br>2. U-shaped E-R relationships: regression coefficient was highest at 0.004 and 0.006 respectively, and MMT is 50–60°F.	1. Under RCP2.6, the annual mortality rate due to temperature change will increase by 2.4% in 2061–2080 relative to baseline, while RCP8.5 predicts a mortality increase up to 14.2%.<br>2. Burden of deaths: RCP8.5>RCP2.6, Rural households>urban households
Sun et al., 2019 [129]	Nanjing	1. Future temperature scenarios: RCPs (4.5, 8.5),<br>2. Population scenarios [a constant scenario and two SSPs scenarios (2, 5)]	2010–2039, 2040–2069, 2070–2099	Heat- and cold-related moralities	1. E-R relationship with a Poisson regression model combined with a DLNM in 2007–2012.<br>Confounders: year for the season and long-term trends, relative humidity, PM_{10}, NO_2, and SO_2, and the day of the week.<br>2. Over a lag up to 14 days, both cold and heat deviating from MMT (24.9 °C) were correlated to additional deaths.	1. Temperature-related deaths will experience minor shifts in periods of 2010–2039, 2040–2069, and 2070–2099 (-5% to -9%). This is due to the counterbalancing effects of the rise in heat-related deaths and the decline in those related to cold.<br>2. Burden of deaths: SSPs>Constant
Huang et al., 2019 [130]	Tianjin	1. Future temperature scenarios: RCPs (2.6, 4.5, 8.5)	2006 to 2011 vs. 2050s and	Heat- and cold-related ischemic heart disease	1. E-R relationship using the DLNM in 2006–2011.<br>Confounders: year for seasonal and	1. The temperature-related YLL per year from IHD increases by 4.5%–38.3% under three scenarios in the 2050s and 2070s

		2. Low-, medium- and high-variant scenarios of population growth<br>3. Adaptation scenarios (Shifting the optimal temperature OT by 1.0°C and 1.2°C in 2050 and 2070)	2070s	(IHD) YLLs in the elderly	long-term trend, day of the week, PM_{10} and humidity.<br>2. Relationship between daily maximum temperature and IHD YLLs along lag 0−15 days: U-shaped with the minimum YLL temperature of 23.6 °C; YLLs of high temperature relative to 23.6 °C was highest at around 80 and YLLs of low temperature was highest at around 85.	compared to the baseline values.<br>2. Burden of deaths: Percent changes of temperature-related YLL in the future vs. baseline: warm season months > other months,<br>Demographic change scenario > no change, Scenario of adaptation<No adaptation
Huang et al., 2018 [131]	Ningbo	1. Future temperature scenarios: RCPs (2.6, 4.5, 8.5)<br>2. Low-, medium- and high-variant scenarios of population growth<br>3. Adaptation scenarios (shifting the OT and shape of temperature-YLL curves)	2011 vs. 2050s, 2070s	Heat- and cold-related cardiovascular disease YLLs in the elderly	1. E-R relationship using the DLNM with Gaussian distribution in 2008–2015. Confounders: year for seasonal and long-term trend, day of the week and humidity.<br>2. Relationship between daily maximum temperature and cardiovascular disease YLLs: U-shaped; YLLs of high temperature relative to the minimum YLL temperature was highest at around 170 and YLLs of low temperature was highest at around 260.	1. The projected YLL attributed to temperature will decrease in the future.<br>2. Burden of deaths: Percent changes of temperature-related YLL in the future vs. baseline: August > other months,<br>Demographic change scenario>no change<br>Scenario of adaptation<No adaptation
Vicedo-Cabrera et al., 2018 [132]	15 locations in China (Global studies including China)	Future temperature scenarios: Scenarios consistent with 1.5, 2, 3, and 4 °C increases in global mean temperature (GMT)	NA	Heat- and cold-related moralities	1. Two-stage analysis derived from a previously published study [133]: location-specific E-R relationship using the DLNM with a quasi-Poisson regression in 1996–2008 in China plus multivariate meta-analysis to pool the E-R relationship. Confounders: time for season, long-term trends, and day of week.<br>2. Take Beijing, China for example, cumulative RR of daily mean temperature across 0–21 days: Inverse J-shaped; Risk rises slowly and linearly for cold temperatures below the MMT (highest RR=1.7). By contrast, risk generally rises quickly and non-linearly at high temperatures (highest RR=1.35).	Under more severe scenarios, the majority of regions may face significantly heightened heat-mortality risks, which are not expected to be offset by the projected reductions in cold-related excess mortality.
Li G & Li, Y et al., 2018 [134]	Tianjin	1. Future temperature scenarios: RCPs (2.6, 4.5, 8.5)<br>2. Adaptation	2008 vs. 2050s and 2070s	Heat- and cold-related cardiovascular disease YLLs	1. E-R relationship using the DLNM in 2006–2011. Confounders: year for seasonal and long-term trend, day of the week and relative humidity, PM_{10}.	1. Without adaptation, net annual YLL decline under RCP2.6 and RCP4.5 and increase under RCP8.5 in 2070s, suggesting that in most cases the rise in YLL from

		scenarios (Shifting the optimal temperature OT by 1.0°C and 1.2°C in 2050 and 2070)			2. Relationship between daily maximum temperature and cardiovascular disease YLLs over lag of 15 days: U-shaped; YLLs of high temperature relative to the minimum YLL temperature was highest at around 60 and YLLs of low temperature was highest at around 255.	cardiovascular mortality could counterbalance the decline in cold-related YLL from cardiovascular mortality.<br>2. Increase of heat-related YLL may be offset by adaptation under RCP2.6 but will not be offset under RCP4.5 and RCP8.5<br>3. Burden of deaths: RCP8.5> RCP4.5>RCP2.6,<br>Scenario of adaptation<No adaptation,<br>Percent change of monthly total temperature-related in the future vs. baseline: summer>other seasons.
Li G & Guo Q, et al., 2018 [135]	Tianjin	1. Future temperature scenarios: RCPs (2.6, 4.5, 8.5)<br>2. Adaptation scenarios (Shifting the optimal temperature OT by 1.0°C and 1.2°C in 2050 and 2070)	2008 vs. 2050s and 2070s	Heat- and cold-related stroke YLLs	1. E-R relationship using the DLNM with Poisson distribution in 2006–2011. Confounders: year for seasonal and long-term trend, day of the week and relative humidity, PM_{10}.<br>2. Relationship between daily maximum temperature and stroke YLLs over lag of 12 days: U-shaped; YLLs of high temperature relative to the minimum YLL temperature was highest at around 210 and YLLs of low temperature was highest at around 520.	1. There is a slight decline in net temperature-related YLL with percent decreases of 0.85–2.2% under 3 scenarios compared with the baseline.<br>2. Projected climate change in the future is expected to result in a rise in heat-related YLL, and this increase may be offset by adaptation under RCP2.6 but will not be offset under RCP4.5 and RCP8.5<br>3. Burden of deaths: RCP8.5> RCP4.5>RCP2.6<br>Scenario of adaptation<No adaptation,<br>Percent change of monthly total temperature-related in the future vs. baseline: summer>other seasons.
Zhang et al., 2018 [136]	Beijing	1. Future temperature scenarios: RCPs (2.6, 4.5, 8.5)<br>2. Population growth scenarios (three SSP scenarios)<br>3. Adaptation scenarios (0% adaptation: when the optimal temperature OT of the baseline climate was used; 100% adaptation: when the OT of the future climate was used; 50% adaptation: the midpoint of the OTs of the above two	2007–2009 vs. 2040–2060, 2060–2080	Heat- and cold-related cardiovascular disease (CVD) moralities	1. E-R relationship using a GLM with a logarithm link and a DLNM in 2007–2009. Confounders: year for seasonal and long-term trend, day of the week and relative humidity, PM_{10}.<br>2. Cumulative RR of daily maximum temperature over lags of 0–14 days: U-shaped; RR was highest at around 1.46 and 1.24 for extreme low and high temperature, respectively, and MMT is 29.2°C.	1. Temperature-related CVD death count is predicted to increase by 3.5–10.2% under various RCPs compared to that during the baseline period, meaning the reduction in cold-related deaths did not offset the increase in that of heat-related deaths.<br>2. Population change scenario > no population change scenario,<br>Adaptation<no adaptation

		types)				
Li et al., 2018b [137]	Beijing	1. Future temperature scenarios: RCPs (4.5, 8.5)<br>2. Low, medium, and high variant scenarios of population growth for China developed by the United Nations	1980s vs. 2020s, 2050s, 2080s.	Heat- and cold-related cause-specific mortalities	1. E-R relationship using a Poisson regression model combined with DLNM in 2008–2013. Confounders: year for seasonal and long-term trend, day of the week.<br>2. Cumulative RR of daily mean temperature over lags of 0–14 days: U- or inversely J- shaped. The MMT was 23.6 °C for ischemic stroke, 24.9 °C for hemorrhagic stroke, and 23.8 °C for acute ischemic heart disease, respectively.	1. Without population change, temperature-related mortality for ischemic stroke will increase significantly (by 100% in 2080s under RCP8.5). Acute ischemic heart disease mortality decreases in 2020s and then increase in 2050s and 2080s under two RCPs (by 20% in 2080s under RCP8.5). Hemorrhagic stroke mortality only increases in 2080s under RCP8.5.<br>2. Burden of deaths: RCP8.5>RCP4.5, High population variation scenario> no population change scenario,<br>For hemorrhagic stroke and acute ischemic heart disease: summer and winter>other seasons; for ischemic stroke: summer>other seasons
Li et al., 2018a [138]	Tianjin	Future temperature scenarios: RCPs (2.6, 4.5, 6.0, 8.5)	2008 vs. 2055, 2090	Heat- and cold-related all-cause YLLs (Temperature and extreme cold and heat)	1. E-R relationship using a GLM combined with DLNM in 2006–2011. Confounders: year for long-term trend, day of the week, and humidity.<br>2. Relationship between daily mean temperature and YLLs across 0–15 days lag: U-shaped; YLLs of high temperature relative to the minimum YLL temperature was highest at around 150 for females and 220 for males. YLLs of low temperature was highest at around 580 for females and 570 for males.	1. For the whole temperature range: Projections for YLL are lower than the baseline levels in scenario where the temperature rise is below 2.0°C, but the temperature-related YLL is expected to increase if future climate change surpasses 2.0°C (RCP6.0 and RCP8.5). This indicates the increased heat-related YLL was not balanced by the decreased cold effect.<br>2. For extreme temperature (>29.1°C and <5.9°C): Estimates of YLL increased under all RCPs.<br>3. Burden of deaths: Males>females
Gasparrini et al., 2017 [139]	15 locations in China (Global studies including China)	Future temperature scenarios: RCPs (2.6, 4.5, 6.0, 8.5)	1990s, 2010s vs. 2030s, 2050s, 2070s, 2090s	Heat- and cold-related moralities	Same as Vicedo-Cabrera et al., 2018 [132]	1. Without any adaptation or population changes, the net excess mortality would decline under lower greenhouse gas emission scenarios (RCP2.6/4.5/6.0). Conversely, under high-emission scenarios RCP8.5, there would be a net increase in temperature-related excess mortality.<br>3. Burden of deaths: RCP8.5>RCP2.6/4.5/6.0

SSP: Shared Socioeconomic Pathways, RCP: representative concentration pathways, NA, not applicable

Table 3S Projected air pollution-related health impacts in China

Study	Scenarios	Time horizon	Health outcomes	Exposure-response (E-R) relationship: methods	Level of pollutants	Health effects
Cheng et al., 2023 [140]	5 scenarios: [a] baseline scenario, clean air scenario, on-time peak-clean air scenario, on-time peak-net zero-clean air, and early peak-net zero-clean air. For all scenarios, SSP1 was selected as the socioeconomic drivers.	2020-2060	$PM_{2.5}$-related deaths	E-R relationships published by Global Burden of Disease (GBD) 2019 [141]: Relative risks were estimated as a function of exposure based on published systematic review, using meta-regression—Bayesian, regularized, trimmed (MR-BRT) fitting.	1. Under on-time peak-net zero-clean air, $PM_{2.5}$ levels are projected to decrease from 33.4 μg/m^3 in 2020 to 24.6 μg/m^3 by 2030 and further to 7.6 μg/m^3 by 2060. Under early peak-net zero-clean air, $PM_{2.5}$ concentration could be reduced further to 22.2 μg/m^3 in 2030.<br>2. Implementing end-of-pipe emission control measures could lead to a nationwide reduction in $PM_{2.5}$ exposure level by 7.4 μg/m^3 in 2030 (clean air vs. baseline); Climate mitigation measures for the 2030-carbon-peak target are estimated to result in a modest reduction of 1.3 μg/m^3 (on-time-peak clean air vs. clean air).	1. Compared to the baseline, early peak-net zero-clean air could prevent 2.62 million premature deaths each year by 2060.<br>2. In 2020–2030, end-of-pipe control policies are expected to be the primary means of reducing deaths associated with $PM_{2.5}$ exposure. After 2030, climate mitigation measures aligned with China's carbon- neutrality goals will take over as the key factor in air quality improvement, providing continuous health benefits till 2060. By 2060, it is estimated that climate mitigation and pollution control measures could avert 1.86 and 0.76 million premature deaths, respectively, in comparison to the baseline.<br>3. Customized regional policies that take into account the energy and industrial structure can lead to more effective improvements in $PM_{2.5}$ air quality and increased health benefits.
Shen et al., 2023 [142]	6 electricity scenarios [b] combining two carbon mitigation targets [business-as-usual scenario (*BAU*), NET] and three air pollution control [baseline (BASE), current legislation (CLE), and maximum feasible reduction (MFR)] in the private vehicle (PV)	2020-2050	$PM_{2.5}$-related deaths	Global Exposure Mortality Model (GEMM) [143]	The ban policy would reduce 0.040–0.104, -0.002–0.013 and -0.009–0.013 million ton of NOx, $PM_{2.5}$ and SO_2 emissions in 2050 under BAU scenarios, respectively. Under the NET scenario, the ban policy would reduce 0.056–0.103, -0.002–0.013 and 0.007–0.013 million ton of NOx, $PM_{2.5}$ and SO_2 emissions in 2050, respectively.	1. The ban policy is projected to prevent 4.4 to 18.6 thousand premature deaths in 2050 under the six scenarios. The health benefits in the NET scenario are less pronounced than in the BAU scenario in 2050 due to the greater use of biomass, which has a higher emission factor, particularly in the Base and CLE scenarios.<br>2. Air pollution mitigation efforts within the electricity sector are expected to lessen disparities between regions.

	sector by banning of new sales of internal combustion engine vehicles					
Mo et al., 2022 [144]	4 scenarios [c] combining two energy structure scenarios (BAU_Energy and 2Deg_Energy) and three end-of-pipe scenarios [No-Further-Control (NFC), CLE, and Maximum Technically Feasible Reduction (MTFR)]: BAU_NFC, BAU, 2Deg, and 2Deg_MTFR	2015 vs. 2015-2050	$PM_{2.5}$- and O_3-related deaths	IER model [145]	1. By 2050, the shift in energy structure is expected to result in a 64%, 75%, and 46% reductions in the emissions of CO_2, NOx, and SO_2 compared to the 2015 levels in Guangdong. Coupled with the strictest end-of-pipe emission control measures, VOCs and primary $PM_{2.5}$ emissions are projected to be significantly lowered by 66% and 78%., respectively<br>2. Under the baseline scenario, there is no anticipated improvement in air quality by 2050. However, in the scenario that includes the transformation of the energy structure, the $PM_{2.5}$ and MDA8 concentrations would drop to 21.7 and 75.5 μg/m^3. With the additional implementation of the most stringent end-of-pipe control measures, $PM_{2.5}$ concentrations are further reduced to 16.5 μg/m^3, but there is no significant improvement in ozone levels.	For the baseline scenario, there is no improvement in public health by 2050 in Guangdong. In contrast, under the scenario with energy structure transformation, total premature deaths are expected to reduce to 35.5 thousand. With the additional implementation of most stringent end-of-pipe control measures, premature deaths would decline to 20.6 thousand.
Shen et al., 2022 [146]	2 scenarios: [d] BAU and China Energy Interconnection Carbon Neutrality (CEICN) scenario	2020-2060	$PM_{2.5}$- and O_3-related deaths	E-R relationships based on previously published studies, and 0.0030 and 0.0003 were taken for all-cause mortality due to $PM_{2.5}$ and O_3, respectively, implying that per 10 μg/m^3 increase in $PM_{2.5}$ or O_3 concentration exposure level resulted in the	1. By 2060, the SO_2, NOx and $PM_{2.5}$ emissions are expected to decrease by 91%, 85%, and 90% respectively compared with the BAU scenario.<br>2. By 2060, the population-weighted $PM_{2.5}$ concentration will be around 10 μg/m^3, down more than 80% from 2015. The mean annual $PM_{2.5}$ concentration will reach Grade I requirements (15 μg/m^3) of the National Ambient Air Quality Standard, but it is still higher than the latest WHO guideline of 5 μg/m^3.	In 2020–2060, approximately 32 million premature deaths due to air pollution can be avoided, of which 30 million and 2 million premature deaths can be respectively due to reductions in $PM_{2.5}$ and O_3 concentrations, with a cumulative loss of life value of 394 trillion CNY.

				relative risks of 1.030 and 1.003.		
Wang et al., 2022 [147]	2 scenarios: [e] *Cr* ("NPS scenario" includes all key proposed policy developments, such as all the commitments of NDCs) and *Ca* ("450 scenario" in China only to meet the requirement of limiting global warming under 2°C, and NPS scenario outside China)	2015s vs. 2030s, 2050s	O_3-related mortality and morbidity and co-benefits on economic costs and health expenditure saving	E-R relationships based on a previously published study [148].	1. O_3 concentration in Northeast and South China is about 60–65 ppbv in the *Cr* scenario and, it will drop significantly by about 1–4 ppbv in 2030 compared with 2015. By 2050, O_3 concentration is comparable to that in 2030 in the *Cr* scenario, but it will show a downward trend of about 3–6 ppbv in more regions, in the *Ca* scenario.<br>2. Other countries in the world also benefit from China's climate mitigation, with reduced O_3 concentrations. Compared with the global geographical averages of 41.6 ppbv and 41.5 ppbv in 2030 and 2050 in the *Cr* scenario, O_3 concentration in 2030 and 2050 in the *Ca* scenario will drop by about 0.04 ppbv and 0.09 ppbv in 2050, respectively.	1. Improved O_3 air quality from the strict policy (*Ca*) could avoid approximately 15,800 and 35,550 premature deaths attributed to ozone globally in 2030 and 2050 compared to *Cr*, of which 78% and 76% occurred in China.<br>2. Avoiding O_3-related deaths could save $3 billion in 2030 and $10 billion in 2050 based on the willingness-to-pay<br>3. Reducing ambient O_3 exposure could also avoid morbidity from asthma attacks, chronic bronchitis, respiratory hospitalizations, and pneumonia. For example, the number of respiratory illnesses associated with bronchodilator use in *Ca* scenario in 2030 would be 25 million fewer than that in the *Cr* scenario, and this number would increase to 54 million by 2050.
Liu et al., 2022 [7]	18 scenarios [f] combining six climate mitigation scenarios (*1.5°C, 2°C, NDC, unambitious, baseline, and 4.5°C*) and three end-of-pipe clean air scenarios (*2015-pollution, current-pollution, and ambitious-pollution*)	2015 vs. 2030, 2050	$PM_{2.5}$-related deaths	E-R relationships published by GBD 2019 [141]	1. Improvement of $PM_{2.5}$ air quality: *ambitious-pollution > current-pollution > 2015-pollution; 1.5°C > 2°C > NDC > unambitious > baseline/4.5°C.*<br>2. Population-weighted concentration $PM_{2.5}$ would decrease under all 12 scenarios combining two clean air scenarios (current-pollution and ambitious-pollution) and all six climate targets—all meeting the national standard (35 μg/m^3) by 2030.<br>3. $PM_{2.5}$ concentrations would be the lowest under the *Ambitious-pollution-1.5°C-goals* (9.0 μg/m^3 by 2050)—the only scenario that could reach the WHO guideline of 10 μg/m^3, and the highest under *2015-pollution-baseline-goals/2015-pollution-4.5°C-goals.*	1. Ageing and improved $PM_{2.5}$ air quality dominated the overall changes in $PM_{2.5}$-related deaths, with ageing becoming increasingly important after 2030.<br>2. Even combining *ambitious-pollution*, *NDC* and looser climate targets would increase or slightly reduce $PM_{2.5}$-related deaths in 2050.<br>3. Under *Ambitious-pollution-1.5°C-goals* and *Ambitious-pollution-2°C-goals*, the improvement in air quality would counteract the impact of ageing to some extent, with the $PM_{2.5}$-related deaths decreasing well below the 2015 baseline values by 2050 (reductions of 0.32–0.55 million deaths compared to *NDC* goals).
Zhang et al., 2021 [149]	4 scenarios [g] combining 2 carbon mitigation scenarios	2015-2035	$PM_{2.5}$-related mortality, morbidity,	GEMM [143]	1. In Sichuan, under BaU_REF, $PM_{2.5}$ concentration is 35 μg/m^3 and 32 μg/m^3 in 2025 and 2035. Under BaU_CLE,	1. Under BaU_REF, $PM_{2.5}$-related premature deaths will be about 150,000 in 2025, and drop to 140,000 in 2035. The

	(baseline scenario (BaU) and 1.5 degree) and 3 end-of-pipe air pollution control measures (reference case with emission control levels fixed at the 2015 level (REF), CLE, and MFR): BaU_REF, BaU_CLE, BaU_MFR, and 1.5deg_MFR		work loss and monetized health co-benefits		the $PM_{2.5}$ concentration would be reduced by 1.3 μg/m^3 in 2025 and 1.7 μg/m^3 in 2035; under BaU_MFR, $PM_{2.5}$ concentration can be reduced by 2.5 μg/m^3 in 2025 and 2.6 μg/m^3 in 2035. Under 1.5deg- MFR, the concentration of $PM_{2.5}$ can be further reduced by 4.5 μg/m^3 in 2025 and 5.4 μg/m^3 in 2035.<br>2. Under BaU, population- weighted $PM_{2.5}$ concentration in 2035 will still exceed the national standard, reaching 64 μg/m^3, and can only drop slightly to 58μg/m^3 under BaU_MFR. Under 1.5deg- MFR, the $PM_{2.5}$ concentration in 2030 can be further reduced to 52 μg/m^3.	implementation of end-of-pipe control policies (BaU_CLE and BaU_MFR) can prevent 4500 and 7200 premature deaths in 2035, respectively. Under 1.5deg_MFR, up to 14 000 excess deaths can be avoided in 2035 (Burden of deaths BaU_REF>BaU_CLE>BaU_MFR>1.5deg_MFR; Ischemic heart disease (IHD)> stroke).<br>2. Under BaU_REF, the per capita incidence risk would decline from 5.4×10^{-8} in 2015 to 4.7×10^{-8} in 2035. Under 1.5 deg-MFR, the reduction of $PM_{2.5}$-related disease risks could be 1.1×10^{-8} by 2035.<br>3. Under BaU_REF, $PM_{2.5}$-related labor loss days will be reduced from 109 million days in 2025 to 97 million days in 2035. Under 1.5deg-MFR, the number of labor loss days avoided reach 20 million in 2035.<br>4. In Sichuan Province, the monetized health co-benefits will reach $23 billion under1.5deg-MFR, exceeding the mitigation cost of 1.7 billion USD of in 2035.
Tong et al., 2021[150]	36 scenarios [h] combining 4 climate-energy targets [SSP2-RCP1.9 (or 1.5 °C), SSP2-RCP2.6 or (2 °C), SSP2-RCP4.5, SSP2-RCP6.0], 3 Retirement strategies (Historical retirement, Performance-based retirement, Early retirement) and 3 Pollution control strengths (Reference, Weak, and Strong)	2030, 2050	Deaths related to the $PM_{2.5}$ emissions from global power plants	GEMM [143]	1. Emissions reductions from global power plants: SSP2-RCP1.9>SSP2-RCP2.6>SSP2-RCP4.5>SSP2-RCP6.0; Early retirement>Historical retirement; Strong>Weak>Reference.<br>2. Under the most stringent scenarios combining RCP1.9, early retirement and strong control, there are huge amount emission reductions (−97% of SO_2, −89% of NO_x and −98% of $PM_{2.5}$ in 2010–2050).	1. $PM_{2.5}$-related deaths can be seen as a function of climate mitigation, pollution control stringencies and retirement strategies ($PM_{2.5}$-related deaths: SSP2-RCP1.9<SSP2-RCP2.6<=SSP2-RCP4.5<=SSP2-RCP6.0; Early retirement<Performance-based retirement<Historical retirement; Strong<Weak<Reference).<br>2. In 2030, deaths related to $PM_{2.5}$ are less sensitive to climate pathway than to pollution control stringency and retirement strategies, while by 2050, differences in $PM_{2.5}$-related deaths across climate scenarios widen, while the impact of retirement strategies on simulated air pollution deaths shrinks.<br>3. By 2050, under the most stringent scenario combining RCP1.9, early

						retirement, and strong control, emissions are significantly reduced (as shown on the left), but the health benefits are not significant (43,600 premature deaths compared with 310,400 in 2010 with a reduction of 86%), which means that future population growth and aging significantly swallow part of health benefits brought by improved air quality.
Zhang et al., 2021 [151]	8 scenarios [i] combining four carbon mitigation target dimension [*reference*, *NDC*, and two *carbon neutrality* scenarios: renewable energies (*RE*)-led and negative emission technologies (*NET*)-led)] and two end-of-pipe air pollution control dimensions [*CLE* and *MFR*]	2020–2060	$PM_{2.5}$ -related premature deaths, morbidity, loss of life expectancy and monetized health impacts based on value of a statistical life and cost of illness	Integrated exposure-response (IER) model [145] was fitted by integrating existing RR information from studies of air pollution, secondhand tobacco smoke, household solid cooking fuel, and active smoking.	1. Improvement of $PM_{2.5}$ air quality: *NET* > *RE* > *NDC* > *Reference*; *MFR* > *CLE*<br>2. By 2035, the national population-weighted average $PM_{2.5}$ concentrations will be 26.31 μg/m³ under the *reference-MFR* scenario and 18.70 μg/m³ under the *RE-MFR* and *NET-MFR* scenarios—both meeting the national standard (35 μg/m^3).<br>3. By 2060, national average $PM_{2.5}$ concentrations will be 6.10 μg/m³ under the *RE-MFR* scenario and 9.57 μg/m³ under the *NET-MFR* scenario, respectively. However, the degree of improvement varies by regions and *RE-MFR* scenario is the only scenario that the air quality of all provinces can reach the WHO guideline by 2060 (10 μg/m^3).	1. The accumulated premature deaths avoided in 2020–60 would be 22–50 million by achieving *carbon neutrality*. Life expectancy in *carbon neutrality* scenarios by 2060 could increase by 0.88–2.80 years per person compared to the *reference* scenario with no carbon mitigation<br>2. Death, bronchitis, hospitalization due to cardiovascular and cerebrovascular disease, emergency room visits, and hospitalization due to respiratory disease attributed to $PM_{2.5}$ acute exposures in 2060 could reduce by 1.02–1.53, 47.76–71.72, 32.56–50.37, 3101.02–4649.22, and 60.52–90.69 million compared with the *reference* scenario.<br>3. If the value of a statistical life is set at more than 12.5 million CNY (39% of the OECD value), the health co-benefits will be greater than emission reduction costs, which means the cost-effectiveness of China's carbon neutrality goal.
Yang et al., 2021 [152]	7 scenarios [j] combining three energy scenarios [*BAU*, emission trading system for carbon scenario (*ETS*)/NDC, and accelerated end-use electrification scenario (*EES*)] and three end-of -pipe pollution emission control scenarios	2015 vs. 2030	$PM_{2.5}$- and O_3-related deaths	For $PM_{2.5}$: IER model [145]<br><br>For O_3: A log-linear relationship was used and the applied RR for cardiovascular (CVD) and respiratory (RESP) diseases	1. Improvement of $PM_{2.5}$ and O_3 air quality: *EES* > *ETS* > *BAU*; *ULC* > *SC* > *BC*. Under *BC*, O_3 concentration would increase or not change.<br>2. In the most stringent scenario (*EES-ULC*), the proportion of the population exposed to $PM_{2.5}$ below national standard of 35 μg/m^3 and WHO guideline of 10 μg/m^3 is expected to double in 2015–2030. In this scenario, $PM_{2.5}$ levels are predicted to drop significantly in most regions, with the national annual average falling by 46%	1. Under most stringent scenario (*EES-ULC*) would reduce $PM_{2.5}$- and O_3-related nationwide mortality by 288.6 thousand or 23% in 2030 than *BAU-BC*, and $PM_{2.5}$- and O_3-related avoided deaths were 235.2 and 53.4 thousand, respectively.<br>2. End-of-pipe pollution controls would account for three quarters of the total avoided mortality.<br>3. Provinces with current high levels of pollution and significant population density are projected to benefit more from the implementation of strategies for

	[basic control scenario (*BC*), strict control scenario (*SC*) and a scenario with fully implementation of ultra-low emission control (*ULC*)]			are 1.03 and 1.12 for per 10 ppb increase of AMDA8.	compared to the least stringent scenario of *BAU-BC*.<br>3. O_3 concentration is anticipated to decrease slightly in *ETS* and *EES*, suggesting that the impact of carbon policy on O_3 is quite limited. The stringent air pollution control measures in the *EES-ULC* scenario have proven more effective in reducing O_3 pollution. with a reduction of over 10% in annual AMDA8 for most regions. Despite these efforts, by 2030, only a small fraction of the population could live in regions meeting WHO guideline for O_3 (100 μg/m^3).<br>4. More prominent reductions in both $PM_{2.5}$ and O_3 concentrations are expected to occur in areas that are currently experiencing heavy pollution.	carbon and pollution control.
Xing et al., 2020 [13]	3 scenarios [k] combining two energy scenarios [*NDC*, Co-Benefit Energy (*CBE*) with enhanced low-carbon policies] and two end-of-pipe control levels [*CLE*, *MFR*]	2015–2035	$PM_{2.5}$ (ambient and household) - and O_3-related deaths and monetized health benefits	IER model [145]	1. The average $PM_{2.5}$ concentration in China's 338 cities is reduced from 49.6 μg/m^3 in 2015, to 21.7 μg/m^3 in 2035 *NDC−MFR*, and 18.4 μg/m^3 in 2035 *CBE−MFR*. In 2035 *CBE−MFR*, all Chinese cities can achieve attainment of the national $PM_{2.5}$ standards (35 μg/m^3), whereas China cannot fully attain the standards under *NDC* scenarios, even with the most stringent end-of-pipe controls (*NDC−MFR*).<br>2. The substantial emission reduction in CBE−MFR can also allow all China's 338 cities at the prefecture or higher level to achieve the O_3 attainment target (i.e., the 90th percentile of daily 8-h maximum concentrations <160 μg/m^3) in 2035.	1. On the national scale, China can expect to prevent approximately 158 thousand premature deaths each year, due to reduction in $PM_{2.5}$ from *NDC−MFR* to *CBE−MFR*, with the most significant impact occurring in three densely populated provinces (Henan, Shandong, and Hebei). The majority of the $PM_{2.5}$-related health benefits, around 77% are from the reduction of household $PM_{2.5}$.<br>2. The reduction in O_3 concentration is estimated to prevent approximately 12 thousand premature deaths annually.<br>3. The implementation of *CBE* policy is projected to yield a substantial human health benefit equivalent to approximately 890 billion Chinese Yuan (CNY), based on a valuation of statistical life (VSL) of 5.24 million CNY. This quantified health benefit is expected to be 8 times greater than the costs incurred in the implementation of policy.
Xie et al., 2020 [153]	2 scenarios: [l]<br>*C0* ("NPS scenario" includes a set of	2015s vs. 2030s,	$PM_{2.5}$-related mortality and morbidity and	IER model [145] and GEMM [143]	1. In the C0 scenario, the $PM_{2.5}$ concentration in the eastern provinces of China is expected to drop by more	1. The improved $PM_{2.5}$ air quality by the stringent policy (*C1*) could reduce premature deaths by 0.37 million

	major policy initiatives, such as all the commitments of NDCs) and *C1* ("450 scenario" in China only to to fulfill the global target of keeping the rise in temperature below 2℃, and NPS scenario outside China)	2050s	co-benefits on economic costs and health expenditure saving		than 20 μg/m^3. The more rigorous policy (*C1*) is projected to further reduce PM2.5 by an additional 2.0 to 4.0 μg/m^3 in the northern and eastern of China in the 2030s, in comparison to *C0*.<br>2. Other nations in East Asian also has co-benefit on decline in $PM_{2.5}$ from China's climate mitigation efforts (approximately 2 to 4 μg/m^3 in 2030s and 4 to 6 μg/m^3 in 2050s in the East Asia, particularly in the Korea and west of Japan).<br>3. The South Asia will also see a reduction in $PM_{2.5}$, but reduction is significantly less than that observed in East Asia.	compared with *C0* in 2050s globally, with 0.36 million from China, 7,600 from South-east Asia, and 740 from India.<br>2. The decrease in ambient $PM_{2.5}$ exposure is expected to prevent a total of 11.3 million cases of morbidity worldwide by 2050s, attributed to asthma attacks and hospital admissions, including 10.8 million from China, 0.2 million from South-east Asia, and 2,000–4,000 from India and Japan.<br>3. In the stringent policy (*C1*), the reduction in ambient $PM_{2.5}$ concentration could save $406 billion and $1206 billion economic costs by 2030s and 2050s globally. In 2050 s, the health expenditure saving will be around $4.1 billion.<br>4. China's efforts in climate mitigation are projected to decrease the per capita work time loss due to $PM_{2.5}$ exposure about 5.6 hours in China and 0.21 hours in Japan by the 2050s.
Chang et al., 2020 [154]	3 scenarios: [m] Reference Scenario (*RS*) without the constraint of peaking CO_2 emissions before 2030; Peaking Scenario (*PS*)/NDC target of peaking CO_2 emissions; Peaking Early Scenario (*PES*)/target of peaking CO_2 emissions by 2025	2030	$PM_{2.5}$-related morbidities due to acute exposure and mortalities due to acute and chronic exposure	IER model [145]	1. The Beijing-Tianjin-Hebei region, Yangtze River Delta, and some areas in the northeast China have been the most severely affected by air pollution, but under the policy scenarios these areas are projected to experience the most significant pollution reduction.<br>2. Compared to *RS*, the annual average $PM_{2.5}$ concentrations are expected to decrease by 4%–20% under *PS* and 7%–27% under *PES* in 2030 at the provincial level	1. In comparison with the *RS*, the projected declines in hospitalization for respiratory disease, cardiovascular and cerebrovascular disease hospitalization, emergency, bronchitis, acute-exposure mortality, and chronic mortality under the *PES* are 341,183, 470,986, 22,498,295, 184,701, 23,610 and 143,815, respectively. These reductions are 57%, 57%, 60%, 57%, 57% and 64% higher than those under the *PS*.<br>2. Health benefits are expected to be more pronounced in provinces where $PM_{2.5}$ concentration reductions are more substantial and population is larger.<br>3. If a higher VSL (US$ 1.92 million) is selected, the net benefits derived from the *PES* can match that of the *PS*.
Li N et al., 2019 [12]	12 scenarios [n] combining four carbon emission reduction pathways [Current National	2015 vs. 2030, 2050	$PM_{2.5}$-related premature deaths and mitigation cost	GBD 2013 integrated exposure-response	1. Improvement of $PM_{2.5}$ air quality: *WBD2* > *NDCCUM* > *NDC2030* > *NPi*; *MFR* > *CLE* > *NFC*.<br>2. $PM_{2.5}$ concentration in 2050 in *NPi-CLE* will be 42 μg/m^3 and will decrease	1. In *NPi-CLE* scenario, the projected premature deaths in 2050 will be 1.61 millionin. This figure is anticipated to decrease by 2%, 5%, and 11% in 2050, in the *NDC2030-CLE*, *NDCCUM-CLE*, and

	Policy (*NPi*, reference), NDC with an emission peak in 2030 (*NDC2030*), NDC with a cumulative emission target (*NDCCUM*), Well below 2 degree (*WBD2*)) and three air pollutant control strategy [(No-Further-Control (*NFC*), *CLE*, MFR)			functions [155]	to 40, 37.45, and 30.9 μg/m^3 in *NDC2030-CLE*, *NDCCUM-CLE*, *WBD2-CLE*, respectively, with the adoption of low-carbon constraints. Further reductions in $PM_{2.5}$ concentration to 18.88 and 16.31 μg/m^3 are anticipated in 2050 in *NDCCUM-MFR* and *WBD2-MFR*, respectively, with the application of the more stringent MFR control strategy.<br>3. All 30 provinces can reach National standard (35 μg/m^3) in 2050 in WBD2-MFR, but only 5 provinces are projected to reach the WHO standard.	*WBD2-CLE*, respectively, corresponding to reductions to 1.57, 1.53, and 1.43 million individuals. Further declines are expected in *NPi-MFR*, *NDC2030-MFR*, *NDCCUM-MFR*, and *WBD2-MFR*, with the number projected to be 1.18, 1.14, 1.08, and 0.98 million, respectively.<br>2. Premature deaths are expected to be higher than the baseline value in 2015, mainly due to the aging population and higher baseline mortality. *WBD2-MFR* is the only scenario that deaths will decrease from 2030 to 2050.<br>3. The decreased cost associated with controlling air pollutant can offset the increased cost from CO_2 reduction measures under the *NDC* target, while this reduction measure is not achievable under the *WBD2* target.
Li M et al., 2019 [11]	4 scenarios: [o]<br>*T*hree policy scenarios that aiming to achieve annual reductions in CO_2 intensity by 3%, 4% or 5% from 2015 to 2030 (*3% Policy*, *4% Policy*/ NDC and 5% *Policy*/2 °C) with No Policy scenario served as the reference.	2030	$PM_{2.5}$- and O_3-related premature deaths	Same as Yang et al., 2021 [152]	1. In the 4% Policy scenario, population-weighted $PM_{2.5}$ reduces by 8.3, 1.7, 0.5, and 0.04 μg/m^3 in China, South Korea, Japan, and the US, respectively, in comparison to No Policy.<br>2. Under the 4% Policy scenario, due to reduction of sulfate, the population-weighted MDA8 ozone concentrations decrease by 1.6, 0.6, 0.5, and 0.2 ppb in China, South Korea, Japan, and the US, respectively, relative to No Policy scenario. Climate policies lead to a decrease in ozone in South China throughout the year but result in an increase in ozone in certain areas of North China in spring and fall, and in most of North China during winter.<br>3. Δ $PM_{2.5}$/Δ ozone and Δ CO_2 is linear, but the regression slope between decreases in ozone and CO_2 emissions is 0.14, a quarter of the slope observed for $PM_{2.5}$, in China.	1. In 4% policy scenario the number of premature deaths avoided due to ozone decline is 0.054 million in China in 2030, which is nearly 60% of those prevented by $PM_{2.5}$ reduction.<br>2. Total avoided premature deaths in South Korea, Japan, and the US are 1200, 3500, and 1900, respectively. In South Korea and Japan, the majority of total avoided deaths are attributed to decrease in $PM_{2.5}$-related mortality, but in the US the reduction in ozone plays a more important role.
Li M et al., 2018	4 scenarios: [p]<br>Three policy scenarios that aiming	2010–2030	$PM_{2.5}$-related premature deaths and	IER model [145]; Further, two	1. By 2030, under the No Policy scenario, the projected national population-weighted annual average	1. In 2030, the No Policy scenario predicts a national tally of over 2.3 million premature deaths. In the 3%

[10]	to achieve annual reductions in CO_2 intensity by 3%, 4% or 5% per year from 2015 to 2030 (3% Policy, 4% Policy/NDC and 5% Policy/2 °C) with No Policy scenario served as the reference.		mitigation cost	additional estimates for exposure-response function were also generated using the Environmental Benefits Mapping and Analysis Program-Community Edition (BenMAP-CE)	$PM_{2.5}$ concentration is 70.1 μg/m^3. Compared to No Policy levels in 2030, $PM_{2.5}$ is projected to decrease by 4.7% to 66.8 μg/m^{3}under the 3% Policy, by 12% to 61.7 μg/m^{3}under the 4% Policy and by 19% to 56.8 μg/m^{3}under the 5% Policy (these levels represent an increase of 21%, 12% and 3.5%, respectively, compared to 2010).<br>2. Central China, notably the regions with high coal consumption, such as Shanxi and Guizhou, experience more significant air quality improvements than other provinces	Policy, 4% Policy and 5% Policy scenarios, $PM_{2.5}$ reduction is projected to avoided approximately 0.036, 0.094 and 0.16 million premature mortalities, respectively.<br>2. Health co-benefits in the 4% Policy scenario surpass the policy costs by 3.7 times when international health valuation estimates are used, while when utilizing recent Chinese health valuation estimates, co- benefits offset 26% of policy costs.
Cai et al., 2018 [19]	2 scenarios [q] that reflect CO_2 and air pollutant emissions: reference (REF) scenario without climate policy and NDC scenario in the electric power generation sector	2010–2050	$PM_{2.5}$-related mortality and implementation costs in electric power generation sector	IER model [145]	1. Achieving the NDC targets for $PM_{2.5}$ concentrations would result in air quality improvement in the majority of regions, with the exception of northwest China.<br>Under the NDC scenario, in 2030 and 2050, about 50% and 86%, respectively, of the national territory would have a declining $PM_{2.5}$ concentration. 83% and 84% of China's territory would meet this target (35 μg/m^3) under the NDC scenario in 2030 and 2050, respectively.<br>2. Certain parts of China, predominantly in the northwest, would have an increasing concentration of $PM_{2.5}$. By 2030, people in northwest China (especially in Gansu, Shaanxi, and Xinjiang provinces) are projected to face deteriorating air quality, but this undesirable situation would diminish by about 2050.	1. NDC can avoid 19,962 premature deaths in 2030 and 368,568 in 2050 in China. The largest health benefit would appear to come from a reduction in stroke incidence, followed by declines in ischaemic heart disease, COPD, and lung cancer.<br>2. It broadly follows the pattern of changes in population-weighted $PM_{2.5}$ concentrations, and they would be unevenly distributed in China. The developed regions in China (especially all eastern regions, and the eastern parts of the south and central regions) would benefit the most from the implementation of NDCs, due to the significant decline in $PM_{2.5}$ concentrations and the population density in those regions. The total incremental premature deaths in northwest China would reach up to 10 083 in 2030.<br>3. By 2030, health benefits would cover 18–62% of costs associated with implementation. In 2050, the overall health benefits are expected to surge, reaching a magnitude of 3–9 times of the implementation costs.

NDC: nationally determined contributions, SSP: Shared Socioeconomic Pathways, RCP: representative concentration pathways. NA, not available.

[a] Baseline: did not account for climate constraints or additional clean air policies in 2020-2060. Clean air scenario: only considered strict end-of-pipe air pollution control measures in 2020-2060. On-time peak-clean air scenario: shared the same air pollution control measures as above but incorporated near-term climate mitigation actions aimed at achieving a

peak in carbon by 2030 with no further additional measures planned after 2060. On-time peak-net zero-clean air: China aimed to reach its carbon emission peak in 2030 and carbon neutrality in 2060, with strict end-of-pipe air pollution control measures adopted in 2020-2060. Early peak-net zero-clean air: enhanced the strength of near-term climate mitigation policies by 20%-50% to encourage an earlier carbon emission peak by 2025. In the last two scenarios, the goal was to achieve net-zero emissions in 2060, accompanied by a land carbon sink of 0.9 Gt CO_2.

[b] BAU scenario does not consider the carbon cap, while the NET scenario is developed under the carbon neutrality goal that the carbon intensity will mainly follow the 2 °C target pathway but reduce more to reach near zero (a 97% reduction) by 2050. BASE scenario assumes that the air pollutant reduction level remains the same as the base year. *CLE*: $PM_{2.5}$ concentration meet China's air quality standard of 35 μg/m³ by 2035—Beautiful China goal); *MFR*: meet the WHO Interim-3 standard of 15 μg/m³ by 2050.

[c] BAU_Energy is established based on the prediction of the World Energy Outlook 2011 (WEO 2011), assumes the energy structure of Guangdong Province will be dominated by traditional fossil fuel energy, representing the future development of Guangdong without any energy transformation measures. 2Deg_Energy scenario complies with the two-degree global warming target. CLE scenario, established by referencing the assessment of the 12th Five-Year Plan legislation, is used in this study as a baseline scenario for end-of- pipe emission control development. NFC scenario assumes that the strength of future end-of-pipe emission control measures will remain at the same level as in 2010–2015; this represents the minimum emission reduction potential. MTFR scenario assumes the implementation of the best available end-of-pipe control measures, ignoring political or economic constraints but considering technical limitations; it reflects the maximum potential emission removal using the technical level of China's 12th Five-Year Plan.

[d] BAU refers to a scenario where carbon emissions on the current trend of energy system development are left continued, in which fossil fuel energy remains dominant in the energy system. In the CEICN scenario, clean energy is extensively exploited, allocated, and used across China. The cumulative carbon dioxide emissions of China from 2018 to 2100 are determined based on the comprehensive principles of equity, efficiency, and capacity, which is one-third of the global carbon budget. In the CEICN scenario, the carbon emission space is set as 200 $GtCO_2$ over 2020–2060, referring to the Paris Agreement and also allocation principles (equity, grandfather, cost-benefit, etc.).

[e and l] NPS scenario and 450 scenario are from the WEO. The 450 scenario represents a scenario to meet the requirement of limiting global warming under 2 °C. WEO2016_NPS scenario is the reference scenario (C0/*Cr*), and WEO2016_450 for China and WEO2016_NPS for other countries is the mitigation scenario (C1/*Ca*).

[f] Climate mitigation scenarios were constructed by combining SSPs and RCPs; climate goal scenarios *1.5°C*, *2°C*, *NDC*, *unambitious*, *baseline*, and *4.5°C* correspond to SSP1-RCP1.9, SSP1-RCP2.6, SSP2-RCP4.5, SSP4-RCP6.0, SSP3-RCP7.0, SSP5-RCP8.5, respectively. NDC: By 2030, China would achieve the carbon peak, and the proportion of non-fossil fuels would rise to 20%. The *2015-pollution* scenario maintains air pollution control at 2015 levels; the *current-pollution* scenario maintains the current policy until 2030 to meet China's air quality standard of 35 μg/m^3 by 2030; the *ambitious-pollution* scenario is based on the implementation of current policies, plus the best available pollution control technologies after 2030 to maximise public health benefits by 2050. *2015-pollution* scenario is broadly consistent with BC below. *Current-pollution* is broadly consistent with *CLE* scenario and SC below; *ambitious-pollution* is broadly consistent with *MFR* scenario and ULC below.

[g] BaU scenario depicts the full attainment of China's initial submitted version of NDC, aiming at a 60%–65% CO_2 emission intensity reduction by 2030 compared with 2005. 1.5 degree scenario depicts a mitigation pathway compatible with the global 1.5 degree climate target and China's recently enhanced NDC targets. CLE scenario follows Sichuan and China's implemented and stated air pollution control policies. MFR scenario represents aggressive air pollution control strategies where all feasible end-of-pipe control measures would be applied to realize the maximum feasible reduction of pollutant emissions.

[h] Climate mitigation scenarios were constructed by combing a fixed socioeconomic development (SSP2) and a range of climate mitigation scenarios that span four levels of climate ambition (RCPs: 1.9, 2.6, 4.5 and 6.0), i.e., SSP2-RCP1.9 (or 1.5 °C), SSP2-RCP2.6 (or 2 °C), SSP2-RCP4.5, SSP2-RCP6.0. Three retirement strategies: Historical retirement allows power plants to operate for their historical expected lifetime before being replaced (40 years); Performance-based retirement prioritizes retirement of the most-polluting plants but slightly reduces the global average lifetime (~33 years); Early retirement again prioritizes retirement of the most-polluting plants but also substantially reduces the average lifetime to ~26 years to adapt stringent climate target. Three levels of pollution control technologies: reference, pollution removal efficiencies of all operating units are kept to the 2018-level; weak, any units whose pollution removal efficiencies below the 2018 average are brought up to that average level of controls; strong: the best-available control technologies are deployed on all units

[i] *Reference*: no carbon emission constraint; *NDC*: the carbon emissions in China peak at 10.8 in 2030 and then gradually drop; Both *net emission* of *RE-led* and *NET- led carbon neutrality* scenario follow a pathway that the carbon emission peaks at 9.7Gt in 2020 and declines to 0 in 2060. See [b] for definitions of CLE and MFR. All scenarios share the same SSP (SSP2).

[j] BAU is based on current legislation and implementation status of energy conservation by the end of 2017. ETS builds a carbon emission trading system covering electricity and cement sectors without considering carbon tax and auction. Consistent with NDC, an emission cap was considered in ETS by generating a reduction in carbon intensity of 62.5% in 2030 compared to 2005, the midpoint of China's 60–65% commitment. As a result, the share of coal-fired electricity generation would decline from 54% in BAU to 45%. EES assumes a 30% growth in the fossil fuel replacement with electricity for transportation, residential burning and industry compared to ETS. BC assumes that all current end-of- pipe pollution control (until the end of 2015) will continue during 2015–2030; SC assumes that more progressive control policies will be released and implemented, i.e., existing policies issued after 2015 were fully considered, including "13th Five-Year Plan for Energy Saving and Emission Reduction", "Three-year Action Plan to Fight Air Pollution", and

specific action plans for NMVOCs and diesel trucks; ULC: assumes that ultra-low emission retrofit will be conducted for major high-polluted industries besides policies in SC e.g., $PM_{2.5}$ for coal-fired industrial boilers and cement meet limits of 10 μg/m^3.

[k] CBE is related to energy conservation (e.g., improvement of energy efficiency) and is designed for air quality attainment only, with no further constraints from the long-term climate goals (i.e., to meet the 2 °C global temperature target set out by Paris Agreement). The total CO_2 emissions in CBE is estimated as 8.8 Gt in 2035. CLE assumes that only the currently existing control policies are in place, including the "Three-Year Action Plan for Winning the Blue Sky War from 2018 to 2020" and the "13th Five-Year Plan during 2015–2020". MFR assumes all of the feasible control policies will be applied to realize the maximal application of end-of-pipe controls.

[m] *RS*: no constraint of peaking CO_2 emissions before 2030; *PS*: Existing policy measures continue, the NDC target of peaking CO_2 emissions by 2030 is realized at the national level, and the national ETS is launched to permit interprovincial trade of CO_2 emissions allowances, and an national average annual carbon intensity reduction rate of around 4% should be realized; PES: Existing policy measures continue, the target of peaking CO_2 emissions by 2025 is realized at the national level, and the national ETS is launched to permit inter- provincial trade of CO_2 emissions allowances, and an national average annual carbon intensity reduction rate of around 5% should be realized.

[n] NPi: current national policy, NDC2030: 11Gt CO_2 emissions in 2030, remain unchanged from 2030 to 2050; NDCCUM: 400Gt Cumulative CO_2 emissions in 2010−2050; WBD2: 290Gt cumulative CO_2 emissions in 2010−2050.

[o and p] The 3% Policy target CO_2 intensity reductions of 3% per year between 2015 and 2030. It simulates a continuation of China's CO_2 intensity reduction commitment prior to the 2015 Paris Agreement; total CO_2 emissions in 2030 are projected to be 13.5 Gt. The 4% Policy target CO_2 intensity reductions of 4% per year between 2015 and 2030 and is consistent with China's recent commitment to halt its rise in CO_2 emissions by 2030 (with projected CO_2 emissions of 11.4 Gt in 2030), achieving a 60–65% reduction in CO_2 intensity by 2030 relative to its 2005 level. The 5% Policy target CO_2 intensity reductions of 5% per year between 2015 and 2030, reduces China's CO_2 intensity to the projected world average in 2030 (with projected emissions of 9.7 Gt in 2030), and is comparable to the scenario that limits the global temperature increase to 2 °C.

[q] NDC: CO_2 emission intensity needs to be about 40% below 2010 emissions by 2030 [roughly 35% below 2030 emissions in REF], and about 90% below 2010 emissions by 2050 [roughly 96% below 2050 emissions in REF]).

References

1. Agache I, Sampath V, Aguilera J, et al. Climate change and global health: A call to more research and more action. *Allergy* 2022; **77**(5): 1389-407.
2. Berrang-Ford L, Sietsma AJ, Callaghan M, et al. Systematic mapping of global research on climate and health: a machine learning review. *Lancet Planet Health* 2021; **5**(8): e514-e25.
3. Cai W, Zhang C, Zhang S, et al. The 2022 China report of the Lancet Countdown on health and climate change: leveraging climate actions for healthy ageing. *Lancet Public Health* 2022; **7**(12): e1073-e90.
4. State Council of China. Opinions on the Complete, Accurate and Comprehensive Implementation of the New Development Concept to Do a Good Job in Carbon Peaking and Carbon Neutrality. 2021. http://www.gov.cn/zhengce/2021-10/24/content_5644613.htm.
5. State Council of China. Carbon Peaking Action Plan by 2030. 2021. http://www.gov.cn/zhengce/content/2021-10/26/content_5644984.htm.
6. IEA. An energy sector roadmap to carbon neutrality in China, IEA, Paris. 2021. https://www.iea.org/reports/an-energy-sector-roadmap-to-carbon-neutrality-in-china.
7. Liu Y, Tong D, Cheng J, et al. Role of climate goals and clean-air policies on reducing future air pollution deaths in China: a modelling study. *The Lancet Planetary Health* 2022; **6**(2): e92-e9.
8. Cheng J, Tong D, Zhang Q, et al. Pathways of China's PM2.5 air quality 2015–2060 in the context of carbon neutrality. *National Science Review* 2021.
9. Yang X, Teng F. Air quality benefit of China's mitigation target to peak its emission by 2030. *Climate Policy* 2018; **18**(1): 99-110.
10. Li M, Zhang D, Li C-T, Mulvaney KM, Selin NE, Karplus VJ. Air quality co-benefits of carbon pricing in China. *Nature Climate Change* 2018; **8**(5): 398-403.
11. Li M, Zhang D, Li C-T, Selin NE, Karplus VJ. Co-benefits of China's climate policy for air quality and human health in China and transboundary regions in 2030. *Environmental Research Letters* 2019.
12. Li N, Chen W, Rafaj P, et al. Air Quality Improvement Co-benefits of Low-Carbon Pathways toward Well Below the 2 °C Climate Target in China. *Environmental Science & Technology* 2019; **53**(10): 5576-84.
13. Xing J, Lu X, Wang S, et al. The quest for improved air quality may push China to continue its CO2 reduction beyond the Paris Commitment. *Proceedings of the National Academy of Sciences* 2020; **117**(47): 29535.
14. Shi X, Zheng Y, Lei Y, et al. Air quality benefits of achieving carbon neutrality in China. *Sci Total Environ* 2021; **795**: 148784.
15. Duan H, Zhou S, Jiang K, et al. Assessing China's efforts to pursue the 1.5°C warming limit. *Science* 2021; **372**(6540): 378-85.
16. Fuhrman J, Clarens AF, McJeon H, et al. The role of negative emissions in meeting China's 2060 carbon neutrality goal. *Oxford Open Climate Change* 2021; **1**(1).
17. Lefebvre D, Fawzy S, Aquije CA, Osman AI, Draper KT, Trabold TA. Biomass residue to carbon dioxide removal: quantifying the global impact of biochar. *Biochar* 2023; **5**(1): 65.
18. Osman AI, Ayati A, Krivoshapkin P, et al. Coordination-driven innovations in low-energy catalytic processes: Advancing sustainability in chemical production. *Coordination Chemistry Reviews* 2024; **514**: 215900.

19. Cai W, Hui J, Wang C, et al. The Lancet Countdown on PM2.5 pollution-related health impacts of China's projected carbon dioxide mitigation in the electric power generation sector under the Paris Agreement: a modelling study. *The Lancet Planetary Health* 2018; **2**(4): e151-e61.
20. Xing R, Hanaoka T, Kanamori Y, Masui T. Achieving China's Intended Nationally Determined Contribution and its co-benefits: Effects of the residential sector. *Journal of Cleaner Production* 2018; **172**: 2964-77.
21. Zhang L, Ruan J, Zhang Z, et al. City-level pathways to carbon peak and neutrality in China. *Cell Reports Sustainability* 2024; **1**(5): 100102.
22. Sun X, Mi Z, Du H, Coffman DM. Impacts of poverty eradication on carbon neutrality in China. *Science Bulletin* 2024; **69**(5): 648-60.
23. Song X, Wang S, Hu Y, et al. Impact of ambient temperature on morbidity and mortality: An overview of reviews. *Science of The Total Environment* 2017; **586**: 241-54.
24. Sun Z, Chen C, Xu D, Li T. Effects of ambient temperature on myocardial infarction: A systematic review and meta-analysis. *Environ Pollut* 2018; **241**: 1106-14.
25. Song X, Jiang L, Zhang D, et al. Impact of short-term exposure to extreme temperatures on diabetes mellitus morbidity and mortality? A systematic review and meta-analysis. *Environ Sci Pollut Res Int* 2021; **28**(41): 58035-49.
26. Moon J. The effect of the heatwave on the morbidity and mortality of diabetes patients; a meta-analysis for the era of the climate crisis. *Environ Res* 2021; **195**: 110762.
27. Lee WS, Kim WS, Lim YH, Hong YC. High Temperatures and Kidney Disease Morbidity: A Systematic Review and Meta-analysis. *J Prev Med Public Health* 2019; **52**(1): 1-13.
28. Liu J, Varghese BM, Hansen A, et al. Hot weather as a risk factor for kidney disease outcomes: A systematic review and meta-analysis of epidemiological evidence. *Sci Total Environ* 2021; **801**: 149806.
29. Zhang Y, Long G, Ding B, et al. The impact of ambient temperature on the incidence of urolithiasis: a systematic review and meta-analysis. *Scand J Work Environ Health* 2020; **46**(2): 117-26.
30. Liu J, Varghese BM, Hansen A, et al. Is there an association between hot weather and poor mental health outcomes? A systematic review and meta-analysis. *Environ Int* 2021; **153**: 106533.
31. Frangione B, Rodríguez Villamizar LA, Lang JJ, et al. Short-term changes in meteorological conditions and suicide: A systematic review and meta-analysis. *Environ Res* 2021: 112230.
32. Gao J, Cheng Q, Duan J, et al. Ambient temperature, sunlight duration, and suicide: A systematic review and meta-analysis. *Sci Total Environ* 2019; **646**: 1021-9.
33. Heo S, Lee W, Bell ML. Suicide and Associations with Air Pollution and Ambient Temperature: A Systematic Review and Meta-Analysis. *Int J Environ Res Public Health* 2021; **18**(14).
34. Thompson R, Hornigold R, Page L, Waite T. Associations between high ambient temperatures and heat waves with mental health outcomes: a systematic review. *Public Health* 2018; **161**: 171-91.
35. Obradovich N, Migliorini R, Mednick SC, Fowler JH. Nighttime temperature and human sleep loss in a changing climate. *Sci Adv* 2017; **3**(5): e1601555.

36. Gong J, Part C, Hajat S. Current and future burdens of heat-related dementia hospital admissions in England. *Environment International* 2022; **159**: 107027.
37. Linares C, Martinez-Martin P, Rodríguez-Blázquez C, Forjaz MJ, Carmona R, Díaz J. Effect of heat waves on morbidity and mortality due to Parkinson's disease in Madrid: A time-series analysis. *Environment International* 2016; **89-90**: 1-6.
38. Chersich MF, Pham MD, Areal A, et al. Associations between high temperatures in pregnancy and risk of preterm birth, low birth weight, and stillbirths: systematic review and meta-analysis. *Bmj* 2020; **371**: m3811.
39. Sexton J, Andrews C, Carruthers S, Kumar S, Flenady V, Lieske S. Systematic review of ambient temperature exposure during pregnancy and stillbirth: Methods and evidence. *Environ Res* 2021; **197**: 111037.
40. Puthota J, Alatorre A, Walsh S, Clemente JC, Malaspina D, Spicer J. Prenatal ambient temperature and risk for schizophrenia. *Schizophr Res* 2021.
41. Khoshhali M, Ebrahimpour K, Shoshtari-Yeganeh B, Kelishadi R. Systematic review and meta-analysis on the association between seasonal variation and gestational diabetes mellitus. *Environ Sci Pollut Res Int* 2021; **28**(40): 55915-24.
42. Zorrilla-Vaca A, Healy RJ, Silva-Medina MM. Revealing the association between cerebrovascular accidents and ambient temperature: a meta-analysis. *Int J Biometeorol* 2017; **61**(5): 821-32.
43. Lu P, Xia G, Tong S, Bell M, Li S, Guo Y. Ambient temperature and hospitalizations for acute kidney injury in Queensland, Australia, 1995–2016. *Environmental Research Letters* 2021; **16**(7): 075007.
44. Falla M, Micarelli A, Hüfner K, Strapazzon G. The Effect of Cold Exposure on Cognitive Performance in Healthy Adults: A Systematic Review. *Int J Environ Res Public Health* 2021; **18**(18).
45. Liang C, Yuan J, Tang X, Kan H, Cai W, Chen J. The influence of humid heat on morbidity of megacity Shanghai in China. *Environ Int* 2024; **183**: 108424.
46. Agarwal S, Qin Y, Shi L, Wei G, Zhu H. Impact of temperature on morbidity: New evidence from China. *Journal of Environmental Economics and Management* 2021; **109**: 102495.
47. Chung SE, Cheong H-K, Park J-H, Kim J-H, Han H. Current and Projected Burden of Disease From High Ambient Temperature in Korea. *Epidemiology* 2017; **28**.
48. Chen K, Breitner S, Wolf K, et al. Projection of Temperature-Related Myocardial Infarction in Augsburg, Germany: Moving on From the Paris Agreement on Climate Change. *Dtsch Arztebl Int* 2019; **116**(31-32): 521-7.
49. Onozuka D, Gasparrini A, Sera F, Hashizume M, Honda Y. Future projections of temperature-related excess out-of-hospital cardiac arrest under climate change scenarios in Japan. *Science of The Total Environment* 2019; **682**: 333-9.
50. Aström C, Orru H, Rocklöv J, Strandberg G, Ebi KL, Forsberg B. Heat-related respiratory hospital admissions in Europe in a changing climate: a health impact assessment. *BMJ Open* 2013; **3**(1).
51. Lin S, Hsu W-H, Van Zutphen AR, Saha S, Luber G, Hwang S-A. Excessive heat and respiratory hospitalizations in New York State: estimating current and future public health burden related to climate change. *Environmental health perspectives* 2012; **120**(11): 1571-7.

52. Brikowski TH, Lotan Y, Pearle MS. Climate-related increase in the prevalence of urolithiasis in the United States. *Proceedings of the National Academy of Sciences* 2008; **105**(28): 9841-6.
53. Kaufman J, Vicedo-Cabrera AM, Tam V, Song L, Coffel E, Tasian G. The impact of heat on kidney stone presentations in South Carolina under two climate change scenarios. *Scientific reports* 2022; **12**(1): 1-7.
54. Graff Zivin J, Song Y, Tang Q, Zhang P. Temperature and high-stakes cognitive performance: Evidence from the national college entrance examination in China. *Journal of Environmental Economics and Management* 2020; **104**: 102365.
55. Deschênes O, Greenstone M, Guryan J. Climate change and birth weight. *American Economic Review* 2009; **99**(2): 211-17.
56. Ngo NS, Horton RM. Climate change and fetal health: The impacts of exposure to extreme temperatures in New York City. *Environmental research* 2016; **144**: 158-64.
57. Hajdu T, Hajdu G. Temperature, climate change, and birth weight: evidence from Hungary. *Population and Environment* 2021; **43**(2): 131-48.
58. Li C, Wang X, Wu X, Liu J, Ji D, Du J. Modeling and projection of dengue fever cases in Guangzhou based on variation of weather factors. *Sci Total Environ* 2017; **605-606**: 867-73.
59. Li C, Lu Y, Liu J, Wu X. Climate change and dengue fever transmission in China: Evidences and challenges. *Science of The Total Environment* 2018; **622-623**: 493-501.
60. Fan J-C, Liu Q-Y. Potential impacts of climate change on dengue fever distribution using RCP scenarios in China. *Advances in Climate Change Research* 2019; **10**(1): 1-8.
61. Davis C, Murphy AK, Bambrick H, et al. A regional suitable conditions index to forecast the impact of climate change on dengue vectorial capacity. *Environ Res* 2021; **195**: 110849.
62. Wu W, Ren H, Lu L. Increasingly expanded future risk of dengue fever in the Pearl River Delta, China. *PLoS Negl Trop Dis* 2021; **15**(9): e0009745.
63. Messina JP, Brady OJ, Golding N, et al. The current and future global distribution and population at risk of dengue. *Nat Microbiol* 2019; **4**(9): 1508-15.
64. Song Y, Ge Y, Wang J, Ren Z, Liao Y, Peng J. Spatial distribution estimation of malaria in northern China and its scenarios in 2020, 2030, 2040 and 2050. *Malar J* 2016; **15**(1): 345.
65. Hundessa S, Williams G, Li S, et al. Projecting potential spatial and temporal changes in the distribution of Plasmodium vivax and Plasmodium falciparum malaria in China with climate change. *Sci Total Environ* 2018; **627**: 1285-93.
66. Ren Z, Wang D, Ma A, et al. Predicting malaria vector distribution under climate change scenarios in China: Challenges for malaria elimination. *Scientific Reports* 2016; **6**(1): 20604.
67. Zhang Y, Bi P, Sun Y, Hiller JE. Projected Years Lost due to Disabilities (YLDs) for bacillary dysentery related to increased temperature in temperate and subtropical cities of China. *J Environ Monit* 2012; **14**(2): 510-6.
68. Li C, Wu X, Ji D, Liu J, Yin J, Guo Z. Climate change impacts the epidemic of dysentery: determining climate risk window, modeling and projection. *Environmental Research Letters* 2019; **14**: 104019.
69. Liu Z, Tong MX, Xiang J, et al. Daily Temperature and Bacillary Dysentery: Estimated Effects, Attributable Risks, and Future Disease Burden in 316 Chinese Cities. *Environmental Health Perspectives* 2020; **128**(5): 057008.
70. Wang Y, Zhang C, Gao J, et al. Spatiotemporal trends of hemorrhagic fever with renal syndrome (HFRS) in China under climate variation. *Proceedings of the National Academy of Sciences* 2024; **121**(4): e2312556121.

71. Zhou XN, Yang GJ, Yang K, et al. Potential impact of climate change on schistosomiasis transmission in China. *Am J Trop Med Hyg* 2008; **78**(2): 188-94.
72. Zhao Q, Li S, Cao W, et al. Modeling the Present and Future Incidence of Pediatric Hand, Foot, and Mouth Disease Associated with Ambient Temperature in Mainland China. *Environ Health Perspect* 2018; **126**(4): 047010.
73. Tjaden NB, Suk JE, Fischer D, Thomas SM, Beierkuhnlein C, Semenza JC. Modelling the effects of global climate change on Chikungunya transmission in the 21(st) century. *Sci Rep* 2017; **7**(1): 3813.
74. Stanke C, Kerac M, Prudhomme C, Medlock J, Murray V. Health effects of drought: a systematic review of the evidence. *PLoS Curr* 2013; **5**.
75. Su B, Huang J, Fischer T, et al. Drought losses in China might double between the 1.5 °C and 2.0 °C warming. *Proc Natl Acad Sci U S A* 2018; **115**(42): 10600-5.
76. Li C, Liu Z, Li W, et al. Projecting future risk of dengue related to hydrometeorological conditions in mainland China under climate change scenarios: a modelling study. *Lancet Planet Health* 2023; **7**(5): e397-e406.
77. Charnley GEC, Kelman I, Green N, Hinsley W, Gaythorpe KAM, Murray KA. Exploring relationships between drought and epidemic cholera in Africa using generalised linear models. *BMC Infect Dis* 2021; **21**(1): 1177.
78. Xu R, Yu P, Abramson MJ, et al. Wildfires, Global Climate Change, and Human Health. *N Engl J Med* 2020; **383**(22): 2173-81.
79. Wu Z, He HS, Keane RE, Zhu Z, Wang Y, Shan Y. Current and future patterns of forest fire occurrence in China. *International Journal of Wildland Fire* 2020; **29**(2): 104-19.
80. Johnston FH, Henderson SB, Chen Y, et al. Estimated global mortality attributable to smoke from landscape fires. *Environ Health Perspect* 2012; **120**(5): 695-701.
81. Chen G, Guo Y, Yue X, et al. Mortality risk attributable to wildfire-related PM2·5 pollution: a global time series study in 749 locations. *The Lancet Planetary Health* 2021; **5**(9): e579-e87.
82. Ford B, Val Martin M, Zelasky S, et al. Future fire impacts on smoke concentrations, visibility, and health in the contiguous United States. *GeoHealth* 2018; **2**(8): 229-47.
83. Liu JC, Mickley LJ, Sulprizio MP, et al. Future respiratory hospital admissions from wildfire smoke under climate change in the Western US. *Environmental Research Letters* 2016; **11**(12): 124018.
84. Neumann JE, Amend M, Anenberg S, et al. Estimating PM2.5-related premature mortality and morbidity associated with future wildfire emissions in the western US. *Environ Res Lett* 2021; **16**(3).
85. Stowell JD, Yang C-E, Fu JS, Scovronick NC, Strickland MJ, Liu Y. Asthma exacerbation due to climate change-induced wildfire smoke in the Western US. *Environmental Research Letters* 2021; **17**(1): 014023.
86. Wang Z, Wu X, Dai W, et al. The Prevalence of Posttraumatic Stress Disorder Among Survivors After a Typhoon or Hurricane: A Systematic Review and Meta-Analysis. *Disaster Med Public Health Prep* 2019; **13**(5-6): 1065-73.
87. Jeffers NK, Glass N. Integrative Review of Pregnancy and Birth Outcomes After Exposure to a Hurricane. *J Obstet Gynecol Neonatal Nurs* 2020; **49**(4): 348-60.
88. Waddell SL, Jayaweera DT, Mirsaeidi M, Beier JC, Kumar N. Perspectives on the Health Effects of Hurricanes: A Review and Challenges. *Int J Environ Res Public Health* 2021; **18**(5).

89. Li C, Zhao Q, Zhao Z, Liu Q, Ma W. The association between tropical cyclones and dengue fever in the Pearl River Delta, China during 2013-2018: A time-stratified case-crossover study. *PLoS Negl Trop Dis* 2021; **15**(9): e0009776.
90. Chen J, Wang Z, Tam C-Y, Lau N-C, Lau D-SD, Mok H-Y. Impacts of climate change on tropical cyclones and induced storm surges in the Pearl River Delta region using pseudo-global-warming method. *Scientific Reports* 2020; **10**(1): 1965.
91. Lin W, Sun Y, Nijhuis S, Wang Z. Scenario-based flood risk assessment for urbanizing deltas using future land-use simulation (FLUS): Guangzhou Metropolitan Area as a case study. *Science of The Total Environment* 2020; **739**: 139899.
92. Deng Z, Xun H, Zhou M, et al. Impacts of tropical cyclones and accompanying precipitation on infectious diarrhea in cyclone landing areas of Zhejiang Province, China. *Int J Environ Res Public Health* 2015; **12**(2): 1054-68.
93. Kang R, Xun H, Zhang Y, et al. Impacts of different grades of tropical cyclones on infectious diarrhea in Guangdong, 2005-2011. *PLoS One* 2015; **10**(6): e0131423.
94. Zheng J, Han W, Jiang B, Ma W, Zhang Y. Infectious Diseases and Tropical Cyclones in Southeast China. *Int J Environ Res Public Health* 2017; **14**(5).
95. Jiao K, Hu W, Ren C, Xu Z, Ma W. Impacts of tropical cyclones and accompanying precipitation and wind velocity on childhood hand, foot and mouth disease in Guangdong Province, China. *Environmental Research* 2019; **173**: 262-9.
96. Cai W, Zhang C, Zhang S, et al. The 2021 China report of the Lancet Countdown on health and climate change: seizing the window of opportunity. *The Lancet Public Health* 2021.
97. Xie X, Weng Y, Cai W. Co-Benefits of CO2 Mitigation for NOX Emission Reduction: A Research Based on the DICE Model. *Sustainability* 2018; **10**(4): 1109.
98. Tong D, Cheng J, Liu Y, et al. Dynamic projection of anthropogenic emissions in China: methodology and 2015–2050 emission pathways under a range of socio-economic, climate policy, and pollution control scenarios. *Atmos Chem Phys* 2020; **20**(9): 5729-57.
99. GBD 2019 Risk Factors Collaborators. Global burden of 87 risk factors in 204 countries and territories, 1990-2019: a systematic analysis for the Global Burden of Disease Study 2019. *Lancet* 2020; **396**(10258): 1223-49.
100. Keswani A, Akselrod H, Anenberg SC. Health and clinical impacts of air pollution and linkages with climate change. *NEJM Evidence* 2022: EVIDra2200068.
101. Anenberg SC, Mohegh A, Goldberg DL, et al. Long-term trends in urban NO₂ concentrations and associated paediatric asthma incidence: estimates from global datasets. *The Lancet Planetary Health* 2022; **6**(1): e49-e58.
102. Dominski FH, Lorenzetti Branco JH, Buonanno G, Stabile L, Gameiro da Silva M, Andrade A. Effects of air pollution on health: A mapping review of systematic reviews and meta-analyses. *Environ Res* 2021; **201**: 111487.
103. Han X, Naeher LP. A review of traffic-related air pollution exposure assessment studies in the developing world. *Environment international* 2006; **32**(1): 106-20.
104. Zhang JJ, Smith KR. Household air pollution from coal and biomass fuels in China: measurements, health impacts, and interventions. *Environ Health Perspect* 2007; **115**(6): 848-55.
105. Fang SC, Cassidy A, Christiani DC. A Systematic Review of Occupational Exposure to Particulate Matter and Cardiovascular Disease. *International Journal of Environmental Research and Public Health* 2010; **7**(4): 1773-806.
106. Fu S, Viard VB, Zhang P. Air Pollution and Manufacturing Firm Productivity: Nationwide Estimates for China. *The Economic Journal* 2021; **131**(640): 3241-73.

107. Li S, Williams G, Guo Y. Health benefits from improved outdoor air quality and intervention in China. *Environmental Pollution* 2016; **214**: 17-25.
108. Xue T, Liu J, Zhang Q, et al. Rapid improvement of PM2.5 pollution and associated health benefits in China during 2013–2017. *Science China Earth Sciences* 2019; **62**(12): 1847-56.
109. Zhang Q, Zheng Y, Tong D, et al. Drivers of improved PM_{2.5} air quality in China from 2013 to 2017. *Proceedings of the National Academy of Sciences* 2019; **116**(49): 24463.
110. Liu Z, Gao S, Cai W, et al. Projections of heat-related excess mortality in China due to climate change, population and aging. *Frontiers of Environmental Science & Engineering* 2023; **17**(11): 132.
111. He C, Kim H, Hashizume M, et al. The effects of night-time warming on mortality burden under future climate change scenarios: a modelling study. *The Lancet Planetary Health* 2022; **6**(8): e648-e57.
112. Chen H, Zhao L, Cheng L, et al. Projections of heatwave-attributable mortality under climate change and future population scenarios in China. *The Lancet Regional Health – Western Pacific* 2022; **28**.
113. He G, Xu Y, Hou Z, et al. The assessment of current mortality burden and future mortality risk attributable to compound hot extremes in China. *Science of The Total Environment* 2021; **777**: 146219.
114. Yang J, Zhou M, Ren Z, et al. Projecting heat-related excess mortality under climate change scenarios in China. *Nature Communications* 2021; **12**(1): 1039.
115. Wang Y, Wang A, Zhai J, et al. Tens of thousands additional deaths annually in cities of China between 1.5 °C and 2.0 °C warming. *Nature Communications* 2019; **10**(1): 3376.
116. Liu T, Ren Z, Zhang Y, et al. Modification Effects of Population Expansion, Ageing, and Adaptation on Heat-Related Mortality Risks Under Different Climate Change Scenarios in Guangzhou, China. *Int J Environ Res Public Health* 2019; **16**(3).
117. Guo Y, Gasparrini A, Li S, et al. Quantifying excess deaths related to heatwaves under climate change scenarios: A multicountry time series modelling study. *PLoS Med* 2018; **15**(7): e1002629.
118. Zhang L, Zhang Z, Ye T, et al. Mortality effects of heat waves vary by age and area: a multi-area study in China. *Environmental Health* 2018; **17**(1): 54.
119. Li Y, Ren T, Kinney PL, Joyner A, Zhang W. Projecting future climate change impacts on heat-related mortality in large urban areas in China. *Environ Res* 2018; **163**: 171-85.
120. Chen K, Horton RM, Bader DA, et al. Impact of climate change on heat-related mortality in Jiangsu Province, China. *Environ Pollut* 2017; **224**: 317-25.
121. Li T, Horton RM, Bader DA, et al. Aging Will Amplify the Heat-related Mortality Risk under a Changing Climate: Projection for the Elderly in Beijing, China. *Scientific Reports* 2016; **6**(1): 28161.
122. Wang P, Tong HW, Lee TC, Goggins WB. Projecting future temperature-related mortality using annual time series data: An example from Hong Kong. *Environmental Research* 2022; **212**: 113351.
123. Sun Z, Wang Q, Chen C, et al. Projection of Temperature-Related Excess Mortality by Integrating Population Adaptability Under Changing Climate - China, 2050s and 2080s. *China CDC Wkly* 2021; **3**(33): 697-701.

124. Zhu D, Zhou Q, Liu M, Bi J. Non-optimum temperature-related mortality burden in China: Addressing the dual influences of climate change and urban heat islands. *Science of The Total Environment* 2021; **782**: 146760.
125. Chen R, Yin P, Wang L, et al. Association between ambient temperature and mortality risk and burden: time series study in 272 main Chinese cities. *Bmj* 2018; **363**: k4306.
126. Gu S, Zhang L, Sun S, et al. Projections of temperature-related cause-specific mortality under climate change scenarios in a coastal city of China. *Environment International* 2020; **143**: 105889.
127. Dong S, Wang C, Han Z, Wang Q. Projecting impacts of temperature and population changes on respiratory disease mortality in Yancheng. *Physics and Chemistry of the Earth, Parts A/B/C* 2020; **117**: 102867.
128. Yu X, Lei X, Wang M. Temperature effects on mortality and household adaptation: Evidence from China. *Journal of Environmental Economics and Management* 2019; **96**: 195-212.
129. Sun QH, Horton RM, Bader DA, Jones B, Zhou L, Li TT. Projections of Temperature-related Non-accidental Mortality in Nanjing, China. *Biomed Environ Sci* 2019; **32**(2): 134-9.
130. Huang J, Zeng Q, Pan X, Guo X, Li G. Projections of the effects of global warming on the disease burden of ischemic heart disease in the elderly in Tianjin, China. *BMC Public Health* 2019; **19**(1): 1465.
131. Huang J, Li G, Liu Y, et al. Projections for temperature-related years of life lost from cardiovascular diseases in the elderly in a Chinese city with typical subtropical climate. *Environmental Research* 2018; **167**: 614-21.
132. Vicedo-Cabrera AM, Guo Y, Sera F, et al. Temperature-related mortality impacts under and beyond Paris Agreement climate change scenarios. *Clim Change* 2018; **150**(3-4): 391-402.
133. Gasparrini A, Guo Y, Hashizume M, et al. Mortality risk attributable to high and low ambient temperature: a multicountry observational study. *Lancet* 2015; **386**(9991): 369-75.
134. Li G, Li Y, Tian L, Guo Q, Pan X. Future temperature-related years of life lost projections for cardiovascular disease in Tianjin, China. *Science of The Total Environment* 2018; **630**: 943-50.
135. Li G, Guo Q, Liu Y, Li Y, Pan X. Projected Temperature-Related Years of Life Lost From Stroke Due To Global Warming in a Temperate Climate City, Asia. *Stroke* 2018; **49**(4): 828-34.
136. Zhang B, Li G, Ma Y, Pan X. Projection of temperature-related mortality due to cardiovascular disease in beijing under different climate change, population, and adaptation scenarios. *Environmental Research* 2018; **162**: 152-9.
137. Li T, Horton RM, Bader DA, Liu F, Sun Q, Kinney PL. Long-term projections of temperature-related mortality risks for ischemic stroke, hemorrhagic stroke, and acute ischemic heart disease under changing climate in Beijing, China. *Environment International* 2018; **112**: 1-9.
138. Li Y, Li G, Zeng Q, Liang F, Pan X. Projecting temperature-related years of life lost under different climate change scenarios in one temperate megacity, China. *Environmental Pollution* 2018; **233**: 1068-75.
139. Gasparrini A, Guo Y, Sera F, et al. Projections of temperature-related excess mortality under climate change scenarios. *The Lancet Planetary Health* 2017; **1**(9): e360-e7.

140. Cheng J, Tong D, Liu Y, et al. A synergistic approach to air pollution control and carbon neutrality in China can avoid millions of premature deaths annually by 2060. *One Earth* 2023; **6**(8): 978-89.
141. GBD 2019 Risk Factors Collaborators. Global burden of 87 risk factors in 204 countries and territories, 1990-2019: a systematic analysis for the Global Burden of Disease Study 2019. *Lancet* 2020; **396**(10258): 1223-49.
142. Shen J, Chen X, Li H, et al. Incorporating Health Cobenefits into Province-Driven Climate Policy: A Case of Banning New Internal Combustion Engine Vehicle Sales in China. *Environmental Science & Technology* 2023; **57**(3): 1214-24.
143. Burnett R, Chen H, Szyszkowicz M, et al. Global estimates of mortality associated with long-term exposure to outdoor fine particulate matter. *Proc Natl Acad Sci U S A* 2018; **115**(38): 9592-7.
144. Mo H, Jiang K, Wang P, Shao M, Wang X. Co-Benefits of Energy Structure Transformation and Pollution Control for Air Quality and Public Health until 2050 in Guangdong, China. *Int J Environ Res Public Health* 2022; **19**(22).
145. Burnett RT, Pope CA, 3rd, Ezzati M, et al. An integrated risk function for estimating the global burden of disease attributable to ambient fine particulate matter exposure. *Environ Health Perspect* 2014; **122**(4): 397-403.
146. Shen J, Cai W, Chen X, et al. Synergies of carbon neutrality, air pollution control, and health improvement — a case study of China energy interconnection scenario. *Global Energy Interconnection* 2022; **5**(5): 531-42.
147. Wang Y, Xie M, Wu Y, et al. Ozone-related Co-benefits of China's Climate mitigation Policy. *Resources, Conservation and Recycling* 2022; **182**: 106288.
148. Turner MC, Jerrett M, Pope CA, 3rd, et al. Long-Term Ozone Exposure and Mortality in a Large Prospective Study. *Am J Respir Crit Care Med* 2016; **193**(10): 1134-42.
149. Zhang S, Wu Y, Liu X, et al. Co-benefits of deep carbon reduction on air quality and health improvement in Sichuan Province of China. *Environmental Research Letters* 2021; **16**(9): 095011.
150. Tong D, Geng G, Zhang Q, et al. Health co-benefits of climate change mitigation depend on strategic power plant retirements and pollution controls. *Nature Climate Change* 2021; **11**(12): 1077-83.
151. Zhang S, An K, Li J, et al. Incorporating health co-benefits into technology pathways to achieve China's 2060 carbon neutrality goal: a modelling study. *The Lancet Planetary Health* 2021; **5**(11): e808-e17.
152. Yang J, Zhao Y, Cao J, Nielsen CP. Co-benefits of carbon and pollution control policies on air quality and health till 2030 in China. *Environment International* 2021; **152**: 106482.
153. Xie Y, Wu Y, Xie M, et al. Health and economic benefit of China’s greenhouse gas mitigation by 2050. *Environmental Research Letters* 2020; **15**(10): 104042.
154. Chang S, Yang X, Zheng H, Wang S, Zhang X. Air quality and health co-benefits of China's national emission trading system. *Applied Energy* 2020; **261**: 114226.
155. GBD 2013 Mortality and Causes of Death Collaborators. Global, regional, and national age-sex specific all-cause and cause-specific mortality for 240 causes of death, 1990-2013: a systematic analysis for the Global Burden of Disease Study 2013. *Lancet* 2015; **385**(9963): 117-71.
